# Targeted DNA methylation by homology-directed repair in mammalian cells. Transcription reshapes methylation on the repaired gene

**DOI:** 10.1093/nar/gkt920

**Published:** 2013-10-09

**Authors:** Annalisa Morano, Tiziana Angrisano, Giusi Russo, Rosaria Landi, Antonio Pezone, Silvia Bartollino, Candida Zuchegna, Federica Babbio, Ian Marc Bonapace, Brittany Allen, Mark T. Muller, Lorenzo Chiariotti, Max E. Gottesman, Antonio Porcellini, Enrico V. Avvedimento

**Affiliations:** ^1^Dipartimento di Medicina Molecolare e Biotecnologie mediche, Istituto di Endocrinologia ed Oncologia Sperimentale del C.N.R., Università Federico II, 80131 Napoli, Italy, ^2^IRCCS CROB, Dipartimento di Oncologia Sperimentale, via Padre Pio, 1 85028 Rionero in Vulture, Italy, ^3^Dipartimento di Medicina e di Scienze della Salute, Università del Molise, 86100 Campobasso, Itay, ^4^Dipartimento di Biologia, Università Federico II, 80126 Napoli, Italy, ^5^Dipartimento di Biologia Strutturale e Funzionale, Università dell’Insubria, Varese 21100, Italy, ^6^Department of Molecular Biology and Microbiology and Biomolecular Science Center, University of Central Florida, 12722 Research Parkway, Orlando, FL 32826, USA and ^7^Institute of Cancer Research, Departments of Microbiology and Biochemistry and Molecular Biophysics, Columbia University Medical Center, New York, NY 10032, USA

## Abstract

We report that homology-directed repair of a DNA double-strand break within a single copy Green Fluorescent Protein (GFP) gene in HeLa cells alters the methylation pattern at the site of recombination. DNA methyl transferase (DNMT)1, DNMT3a and two proteins that regulate methylation, Np95 and GADD45A, are recruited to the site of repair and are responsible for selective methylation of the promoter-distal segment of the repaired DNA. The initial methylation pattern of the locus is modified in a transcription-dependent fashion during the 15–20 days following repair, at which time no further changes in the methylation pattern occur. The variation in DNA modification generates stable clones with wide ranges of GFP expression. Collectively, our data indicate that somatic DNA methylation follows homologous repair and is subjected to remodeling by local transcription in a discrete time window during and after the damage. We propose that DNA methylation of repaired genes represents a DNA damage code and is source of variation of gene expression.

## INTRODUCTION

DNA methylation is a feature of higher eukaryote genomes. It is thought to help organize large segments of noncoding DNA in heterochromatin and to contribute to genome stability (1). DNA methylation is critical during development in plants and mammals. In somatic cells, patterns of methylated CpGs are transmitted to daughter cells with high fidelity (2,3). Aberrant methylation, both hyper- and hypo-methylation, has been found in cancer cells (4).

There are two patterns of DNA methylation: (i) Stable methylation, which is the basis of imprinting, is inherited in a sex-specific fashion and is invariant among individuals and cell types. Loss or modification of stable methylation results in significant phenotypic and genetic alterations. (ii) Unstable or metastable methylation, which is variable among individuals and cell types.

Despite numerous analyses of the methylation profiles of single chromosomes, the regulation of DNA methylation is largely unknown. Somatic DNA methylation is associated with gene silencing and heterochromatin formation and is neither sequence- nor cell-specific.

We are investigating the nature of somatic DNA methylation and its link to gene silencing during neoplastic progression (5,6). Since formation of DNA double-strand breaks (DSBs) and activation of DNA damage checkpoints may precede genomic instability (7) and DNA methylation and gene instability appear to be linked in cancer (8), we speculated that DNA methylation was associated with DNA damage and repair.

We previously reported that homology-directed repair (HDR) modifies the methylation pattern of the repaired DNA (9). This was demonstrated using a system pioneered by Jasin (10,11), in which recombination between partial duplications of a chromosomal Green Fluorescent Protein (GFP) gene is initiated by a specific DSB in one copy. The unique DSB is generated by cleavage with the meganuclease I-SceI, which does not cleave the eukaryotic genome. The DSB is repeatedly formed and repaired, until the *I-SceI* site is lost by homologous or nonhomologous repair or depletion of I-SceI enzyme. Recombination products can be detected by direct analysis of the DNA flanking the DSB or by the appearance of functional GFP (9).

Two cell types are generated after recombination: clones expressing high levels of GFP and clones expressing low levels of GFP, referred to as H and L clones, respectively. Relative to the parental gene, the repaired GFP is hypomethylated in H clones and hypermethylated in L clones. The altered methylation pattern is largely restricted to a segment just 3′ to the DSB. Hypermethylation of this tract significantly reduces transcription, although it is 2000 bp distant from the strong cytomegalovirus (CMV) promoter that drives GFP expression (9,12). The ratio between L and H clones is ∼1–2 or 1–4, depending on the insertion site of the GFP reporter. These experiments were performed in mouse embryonic (ES) or human cancer (Hela) cells. HDR-induced methylation was dependent on DNA methyl transferase I (DNMT1). Furthermore, methylation induced by HDR was independent of the methylation status of the converting template (9). These data, taken together, argue for a cause–effect relationship between DNA damage-repair and DNA methylation.

The link between DNA damage, repair and de novo methylation has been confirmed by other studies (13–15). We also note that genome wide surveys show that imprinted sites are historical recombination hot spots, reinforcing our conclusion and that of other workers, that DNA methylation marks the site of DNA recombination (16,17).

We report here that methylation induced by HDR is influenced by recruitment of Np95 and GADD45a to the DSB and that DNMT3a is also active at the DSB. We also show that methylation is reduced by transcription of the repaired region.

## MATERIALS AND METHODS

### Cell culture, transfections and plasmids

HeLa cells lines were cultured at 37°C in 5% CO_2_ in RPMI medium supplemented with 10% fetal bovine serum (Invitrogen), 1% penicillin-streptomycin, and 2 mM glutamine.

HeLa-pDRGFP cells were obtained by transfection of HeLa cells with the pDRGFP plasmid. Briefly: 5 × 10^6^ cells were seeded in a 100 mm dish and transfected with lipofectamine as recommended by the manufacturer (Invitrogen) with 2 µg of linearized pDRGFP plasmid and selected in the presence of puromycin (2 micrograms/ml). Four clones were isolated and expanded, the remaining clones were screened for single pDRGFP insertion by quantitative Polymerase Chain Reaction (qPCR) [supporting information in (9)] and pooled (∼200 clones with a pDRGFP copy number ranging from 0.8 to 1,2 copies/genome). Clone 3 is the same clone 3 described in (9); clone 4 is a subclone of the clone 2 assayed also by Southern Blot (9). 10^6^ puromycin-resistant cells were transient transfected by electroporation with 2.5 µg of plasmid DNAs and/or small interfering RNA (siRNA) (200 nM) as indicated in the Figures. After transfection cells were seeded at 3 × 10^5^ cells per 60 mm dish, 24 h post-transfection, cells were treated and harvested as described in figures. Pools of clones were generated in three independent transfections and frozen in aliquots. Transient transfections with I-SceI were carried at different times of culture after the primary transfection. Transfection efficiency was measured by assaying β-galactosidase activity of an included pSVβGal vector (Promega). Normalization by fluorescent-activated cell sorter (FACS) was performed using antibodies to β-gal or pCMV-DsRed-Express (Clontech). pEGFP (Clontech) was used as GFP control vector. The structure of the pDRGFP and other plasmids are described in the supplementary data (Suplementary Methods and Supplementary Figure S12).

### Nucleic acid extraction and quantitative reverse Transcription Polymerase Chain Reaction, qPCR and PCR

Total RNA was extracted using Triazol (Gibco/Invitrogen). Genomic DNA extraction was performed as described in (9). cDNA was synthesized in a 20 µl reaction volume containing 2 µg of total RNA, four units of Omniscript Reverse Transcriptase (Qiagen), and 1 µl random hexamer (20 ng/µl) (Invitrogen). mRNA was reverse-transcribed for 1 h at 37°C, and the reaction was heat inactivated for 10 min at 70°C. The products were stored at −20°C until use. Amplifications were performed in 20 µl reaction mixture containing 2 µl of synthesized cDNA product or 0.1 µg of genomic DNA, 2 µl of 10X PCR buffer, 1.5 mM MgCl_2_, 0.5 mM dNTP, 1.25 unit of Taq polymerase (Roche), and 0.2 µM of each primer on a TC3000G thermocycler (Bibby Scientific Italia). The number of cycles was selected and validated by running several control reactions and determining the linear range of the reaction. 15 µl of the PCR products were applied to a 1.2% agarose gel and visualized by ethidium bromide staining. Densitometric analysis was performed using a phosphoimager. Each point was determined in at least three independent reactions. Quantitative reverse Transcription Polymerase Chain Reaction (qRT-PCR) and qPCR were performed three times in six replicates on a 7500 Real Time-PCR on DNA template (RT-PCR) System (Applied Biosystems) using the SYBR Green-detection system (FS Universal SYBR Green MasterRox/Roche Applied Science). The complete list of oligonucleotides is reported in Supplementary Table S1.

### FACS analysis

**Figure 1.**
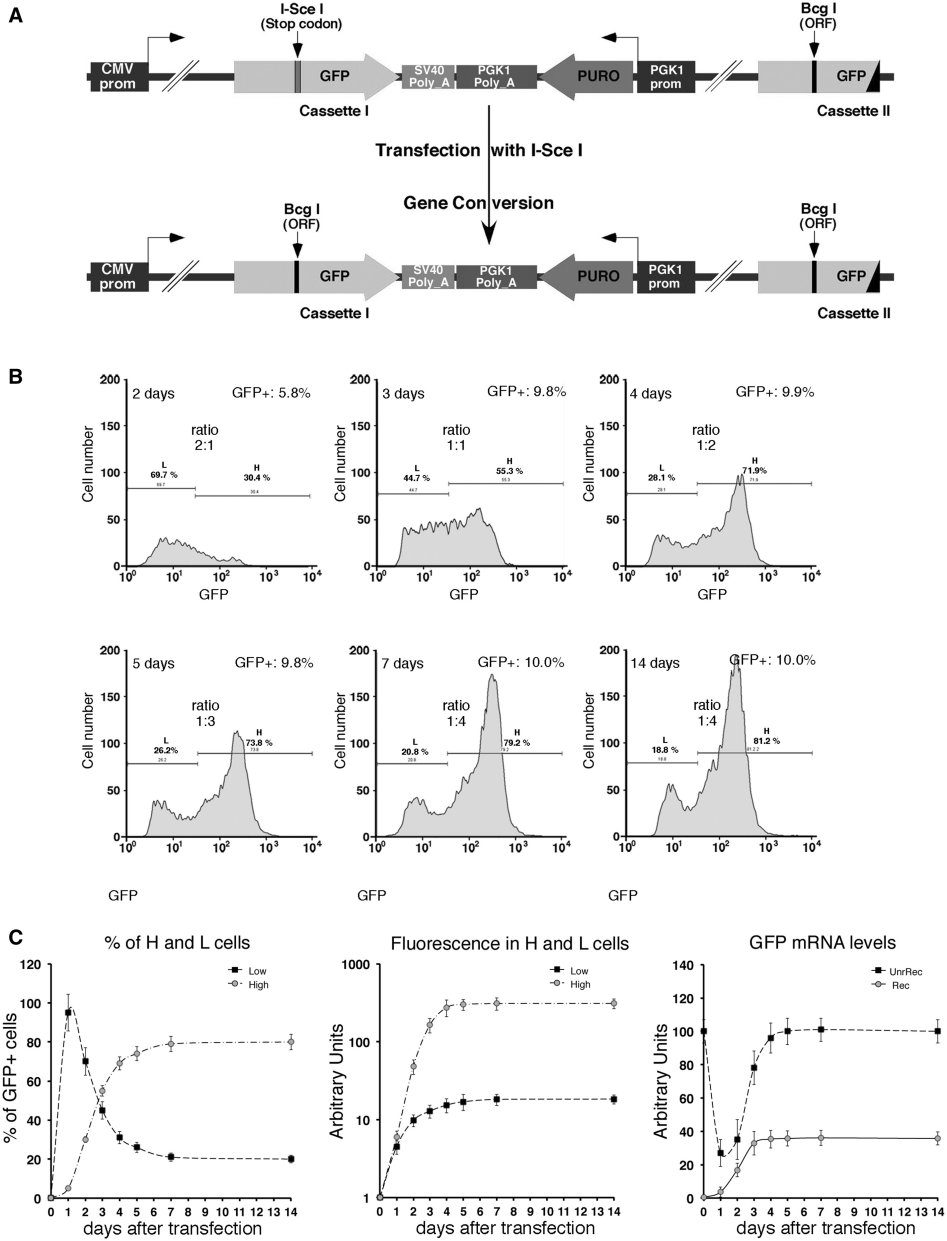
HDR generates high and low GFP-expressing clones. (**A**) Structure of the integrated tester DRGFP plasmid before and after repair. The structure of the plasmid (10,11) has been verified by sequence analysis. The boxes and arrows with different grayscales represent the structural elements of the integrated nonrecombinant (upper) and recombinant (lower) plasmid. The conversion of the *I-SceI* to *BcgI* restriction site marks the gene conversion event driven by the copy of GFP gene located at the 3′ end of DRGFP (cassette II). (**B**) Generation and accumulation of high (H) and low (L) expressor cells following homologous repair. Kinetics of L and H clones accumulation. Cells containing a single copy of DRGFP (clones 3 and 4, see ‘Materials and Methods’ section) or pool of clones (shown here), characterized as described in ‘Materials and Methods’ section, were transfected with I-SceI and subjected to FACS analysis at the times indicated. GFP positive (GFP^+^) cells were identified by the R1 gate (see Supplementary Figure S3A) on a bivariate plot (FL1H versus FL2H) after I-SceI transfection. A representative experiment, displaying the L and H cells is shown. Each panel shows (i) the days after I-SceI transfection; (ii) total GFP positive cells (%); (iii) the range gates used to discriminate H and L cells; (iv) the ratio L/H, which reached a plateau 7–14 days after I-SceI transfection. Panel (**C**): the number (percent of total GFP^+^ cells, left) and the fluorescence intensity (mean, center) of H and L cells derived from clones (not shown here) or pool of clones, based on at least five independent experiments. After 7–14 days, the L/H ratio and the intensity of L and H peaks stabilize. CMV–EGFP transfected cells, as control lines, display a single fluorescence peak (9). The right panel shows the relative levels, normalized to 18 S RNA, of nonrecombinant (UnRec) and recombinant (Rec) GFP mRNA after I-SceI transfection (see ‘Materials and Methods’ section).

HeLa-DRGFP cells were harvested and resuspended in 500 µl of phosphate buffered saline (PBS) at density of 10^6^ cells/ml. Cell viability was assessed by propidium iodide (PI) staining. Cytofluorimetric analysis was performed on a 9600 Cyan System (Dako Cytometrix) or FACScan Flow Cytometer (Becton Dickinson, Franklin Lakes, NJ, USA). PI positive cells were excluded from the analysis by gating the PI-negative cells on a FSC-Linear versus FL2H-Log plot. GFP^+^ cells were identified by using a gate (R1 in Supplementary Figure S3A) on a FL1H-Log versus Fl2H-Log plot after sample compensation for FL1 versus FL2 channels. L and H cells were identified on FL1H Histogram of the R1-gated cells with two range-gate, as shown in Figure 1. The same gate was used for all cytofluorimetric determinations.

Cell cycle analysis was carried out by FACS: 1 × 10^6^ cells were resuspended in 1 ml of PBS and fixed 10 ml of ice-cold 70% ethanol. Afther 3 h, the cells were washed and stained for 30 min at room temperature with 0.1% Triton X100, 0.2 mg/ml Dnase-free RnaseA, 20 µg/ml PI. Fluorescence was evalued by FACS and analyzed by ModFit LT 2.0 (Verity Software House, Topsham, ME, USA).

Population comparison was performed using the Population Comparison module of the FlowJo software (Tree Star, Inc., Ashland, OR). Difference in fluorescence intensity (mean) was determined using the matched pairs Student’s *t* test.

### Bisulfite DNA preparation, PCR and sequence analysis

Sodium bisulfite analysis was carried out on purified genomic DNA and on ‘chromatinized’ DNA. The full list of the buffer formulation is reported in the Supplementary Methods (Buffers Formulation). Chromatinized DNA was obtained as follows: 10^7^ cells were fixed at 4°C temperature with 1% formaldehyde for 3 min. The reaction was stopped with glycine to a final concentration of 125 mM. Nuclei were isolated and permeabilized by incubating cells for 20 min in Buffer A, 20 min in Buffer B and then resuspended in Buffer C (see Buffers Formulation in Supplementary Methods). Nuclei or purified genomic DNA was heat denaturated (96°C for 10 min) incubated in a fresh solution containing 5 M sodium bisulfite and 20 mM hydroquinone and incubated at 37°C for 18 h. The cross-link was reversed, and proteins were digested with proteinase K (50 µg/ml at 55°C for 2 h, and then at 65°C overnight). DNA was purified using a Wizard genomic purification kit (Promega), and then disulphonated by incubation for 15 min with NaOH to a final concentration of 0.3 M, neutralized with ammonium acetate to a final concentration of 3 M, and purified by ethanol precipitation. DNA was amplified by PCR using primers, listed in the Supplementary Table S1, using Taq polymerase, which is able to copy deoxyuridine, cloned in TOPO TA vector (Invitrogen), and sequenced with the M13 reverse primers.

### Chromatin Immunoprecipitation

Cells were transfected and/or treated as indicated in the legends of the figures. The cells (∼1 × 10^6^) were fixed by adding formaldehyde directly in the culture medium to a final concentration of 1% for 10 min at room temperature and washed twice using ice cold PBS containing 1× protease inhibitor cocktail (Roche Applied Science) and 1 mM Phenylmethylsulfonyl Fluoride (PMSF). Fixed cells were harvested and the pellet was resuspended in 200 µl of sodium dodecyl sulphate Lysis Buffer (ChIP Assay Kit/Upstate). After 10 min incubation on ice, the lysates were sonicated to shear DNA to 300-and 1000-bp fragments. Sonicated samples were centrifuged and supernatants duluted 10-fold in the ChIP Dilution Buffer (ChIP Assay Kit/Upstate). An aliquot (1/50) of sheared chromatin was further treated with proteinase K, phenol/chloroform extracted and precipitated to determine DNA concentration and shearing efficiency (input DNA). The chromatin immunoprecipitation (ChIP) reaction was set up according to the manufacturer’s instructions. Briefly, the sheared chromatin was precleard for 2 h with 20 µl of protein-A or protein-G agarose (Upstate) and 2 µg of nonimmune IgG (New England Biolabs). Precleared chromatin was divided in two aliquots and incubated at 4°C for 16 h with 20 µl of protein-A/G agarose and 2 µg of the specific antibody (Np95, generated and characterizated by IM Bonapace; RNA Pol II from Upstate cat. # 05-623; DNMT1, DNMT3a and DNMT3b from Abcam, cat. # ab-13537, ab-2850 and ab-2851, respectively) and nonimmune IgG respectively. Agarose beads were washed with wash buffers according to the manufacturer’s instructions and immunoprecipitated DNA was recovered and subjected to qPCR using the primers indicated in the legend of the specific figures and in Supplementary Table S1.

### Methylated DNA immunoprecipitation

Cells were transfected and/or treated as indicated in the legend of the figures. The cells (∼5 × 10^6^) were harvested and genomic DNA extracted as described above. Ten micrograms of total genomic DNA were digested in 200 µl for 16 h with restriction endonuclease mix containing 30 U each of Eco RI, Bam HI, Hind III, XbaI, Sal I (Roche Applied Science), phenol/chloroform extracted, ethanol precipitated and resuspended in 50 µl of Tris-HCl/EDTA buffer (10 mM Tris-HCl pH 7.8, 1 mM EDTA) (TE) buffer. An aliquot (1/10) of digested DNA was used as input to determine the DNA concentration and digestion efficiency. Methylated DNA immunoprecipitation (MEDIP) was performed essentially as described (18) except that 2 µg of antibody specific for 5mC (Abcam cat. # ab-124936) were used to precipitate methylated DNA from 5 µg of total genomic DNA. H19 and UE2B were used to control in each experiment the efficiency of 5mC immunoprecipitation; the CpG island located to 5′ end of human beta-actin was used as undamaged transcribed DNA gene control.

### Statistical analysis

All data are presented as mean ± standard deviation in at least three experiments in triplicate (n ≥ 9). Statistical significance between groups was determined using Student’s *t* test (matched pairs test or unmatched test were used as indicated in figure legends). Hierarchical clustering (Ward's criterion) analysis was performed using the *JMP Statistical Discovery™* software by SAS, Statistical Analysis Software. Sequence analysis and alignments were performed using MegAlign software (a module of the Lasergene Software Suite for sequence analysis by DNASTAR) for MacOSX.

## RESULTS

### Repair-induced methylation at the 3′ end of a DSB

The system we use to study DNA methylation induced by damage and repair relies on a single-copy integrated plasmid (DRGFP), which contains two inactive versions of GFP. Introduction of a DSB in one copy of the gene (cassette I) by expressing the nuclease I-SceI, generates a functional GFP only in cells in which the second copy of GFP (cassette II) provides the template to repair the DSB (10,11) (Figure 1A). Homologous repair both in pools and single clones generates cells expressing low (L clones) or high levels (H clones) of GFP. These clones can be tracked by FACS analysis, using bivariate plots and gating strategies.

The integrated DRGFP undergoes several cycles of cutting and resealing until the *I-SceI* site is lost by nonhomologous end joining (NHEJ) or homology-dependent repair (HDR). We defined the time window of HDR by monitoring the appearance of recombinant GFP DNA in the population of cells transiently expressing I-SceI. We also measured the levels of I-SceI protein in transfected cells to estimate the period of enzymatic cleavage. Supplementary Figure S1A shows that the levels of recombinant GFP reached a plateau 3 days after transfection with I-SceI. The enzyme accumulated between 12 and 24 h and progressively disappeared 48–72 h after transfection. The estimated half-life of I-SceI protein was between 12 and 24 h (Supplementary Figure S1B).

Having established that the bulk of repair activity occurred in 3 days, we monitored the appearance and stabilization of L and H clones during and after HDR (9). Figure 1B shows the accumulation of L and H cells after exposure to I-SceI in a pool of HeLa clones as well as in single insertion clones carrying DRGFP inserts at different loci (see the legend of Figure 1B). Three days after I-SceI transfection, when HDR was almost complete, L and H cells accumulated in a 1:1 ratio (Figure 1B). We have used time-lapse microscopy to monitor GFP appearance during 30 h after I-SceI induction. The Supplementary Movie shows the I and II/III cycles (relative to GFP expression) during repair and the appearance of H and L cells from single repair events. In the I cycle, H and L cells are generated; in the II/III cycle (H-H and L-L), the phenotypes are stably propagated. Eventually, the ratio L/H cells changes as a function of time, until day 7 when the L to H ratio stabilized at 1:4 (Figure 1B). No further change was detected after numerous subsequent passages, and no new GFP clones appeared (data not shown). Note that this shift to high GFP-expressing cells occured after DSB repair, and therefore represents an inherited epigenetic process.

The drift toward H clones is detailed in Figure 1C. This figure also shows the levels of GFP mRNA as a function of time after transfection with the I-SceI plasmid. The changes in GFP mRNA concentrations correlate well with the fluorescence measurements that reflect GFP expression. We wondered if the time-dependent epigenetic changes were related to transcription of the GFP gene. This notion was tested by adding α-amanitin during repair and following the appearance of L and H clones. α-Amanitin inhibits translocation of elongating RNA polymerase II (Pol II) and increases the concentration of the polymerase on transcribed genes (19).

**Figure 2.**
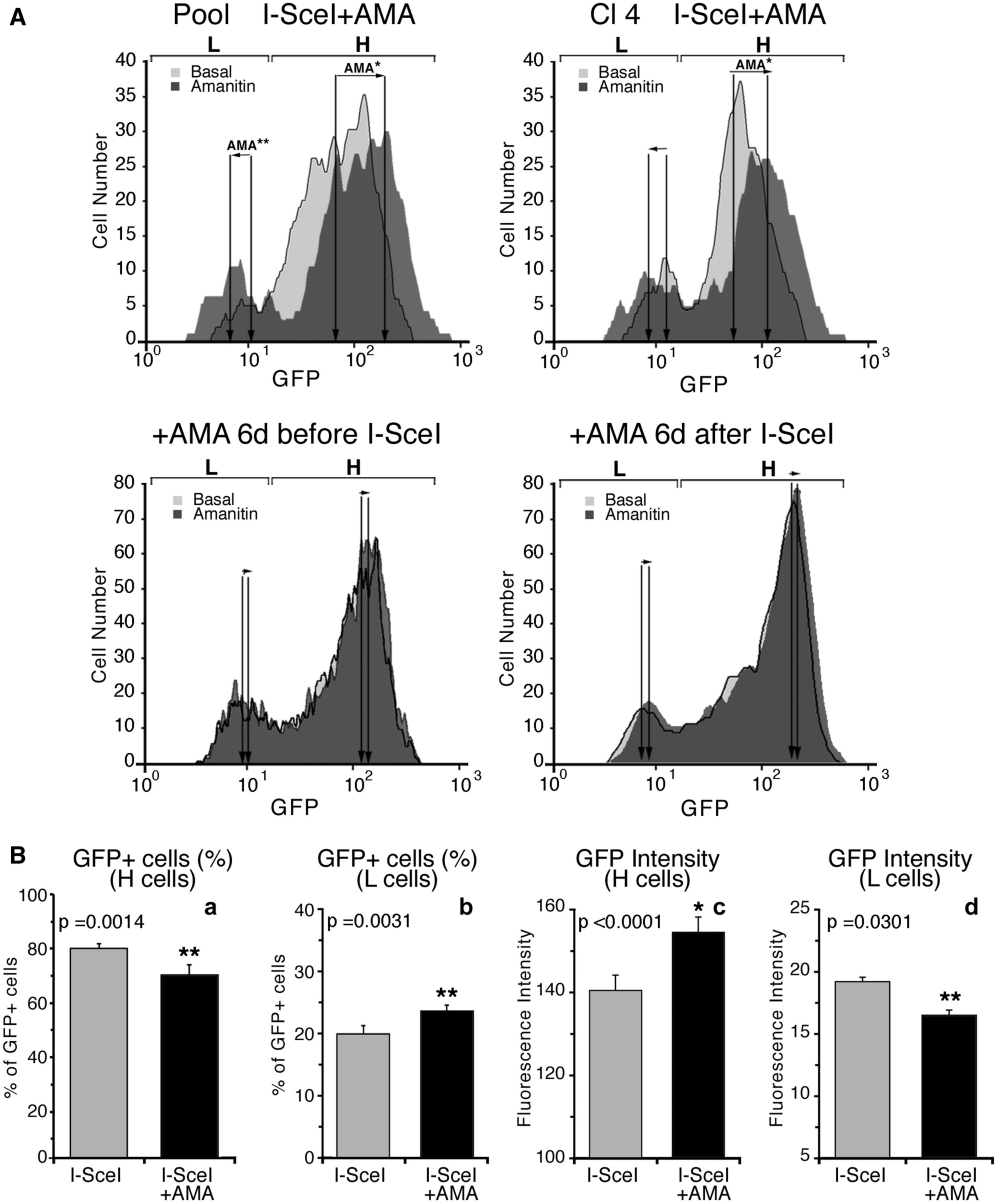
Synchronization of transcription by α-amanitin during repair amplifies and consolidates L and H clones. (**A**) Cytofluorimetric analysis. Cells were exposed to α-amanitin before, during or after I-SceI transfection as indicated on the top of each panel. A pool of HeLa DRGFP cells or a clone carrying a single insert were transfected with I-SceI expression vector, and 24 h later, an aliquot was exposed for 24 h to 2.5 µM α-amanitin. The cells were washed and cultured in normal medium for 5 days, when FACS analysis was carried out (day 7 after I-SceI transfection). The fluorescence plots of GFP positive cells (overlay of the histograms of RI gates, see Supplementary Figure S3) are shown. L and H represent the range gates to identify high and low expressors, respectively. The arrows, indicated by AMA, represent the shift of the mean fluorescence after α-amanitin treatment. Differences between treatments were tested for statistical significance using Student’s matched pairs *t* test: *P < 0.001, **P < 0.05. Under these conditions, α-amanitin did not affect cell survival or growth rate. Five days after 24-h 2.5 -µM α-amanitin treatment, transcription of several housekeeping genes was similar to untreated controls. The changes of GFP expression following the short treatment(s) with the drug during repair (24 h after I-SceI transfection) were stable for up 3 months in culture. (**B**) Statistical analysis derived from 28 independent experiments, in which DRGFP cells were exposed to α-amanitin during repair as indicated above. The panel shows the statistical significance of the means ( ± SD). Differences between treatments were tested for statistical significance using Student’s matched pairs *t* test: *P < 0.001, **P < 0.05.

The pool of DRGFP clones, as well as one clone (Cl4), was transfected with I-SceI, and after 24 h, exposed to α-amanitin for 24 h. Five days later (day 7 after I-SceI transfection), GFP expression was analyzed by cytofluorimetry. Exposure of the cells to the drug did not influence the rate of recombination (Supplementary Figure S2A). As expected, it significantly enriched GFP chromatin with Pol II molecules (Supplementary Figure S2B). Figure 2A and Supplementary Figure S3 show that α-amanitin treatment of pooled cells (or clone 4) shifted the populations of L and H classes in opposite directions (see arrows AMA): L and H cells displayed on the average, lower or higher fluorescence intensity, respectively. Exposure to α-amanitin 6 days before transfection with I-SceI or 6 days after did not affect the distribution of L and H clones (Figure 2A and Supplementary Figure S3A). Statistical analysis of the data of 28 independent experiments in which α-amanitin was added during recombination to pools or single clones indicates that the results are reproducible (Figure 2B and Supplementary Figure S3B). Quantitative analysis of GFP fluorescence in H and L cells exposed to α-amanitin during repair reveals that the fraction of L cells increased and that the GFP expression in these cells was markedly reduced. Conversely, the H cell fraction decreased, but the intensity of the fluorescent signal in these cells was enhanced (Figure 2B). We hypothesize that transient stalling of Pol II induced by α-amanitin during repair, increased GFP methylation, yielding higher numbers of L clones and reducing the fraction of H clones.

**Figure 3.**
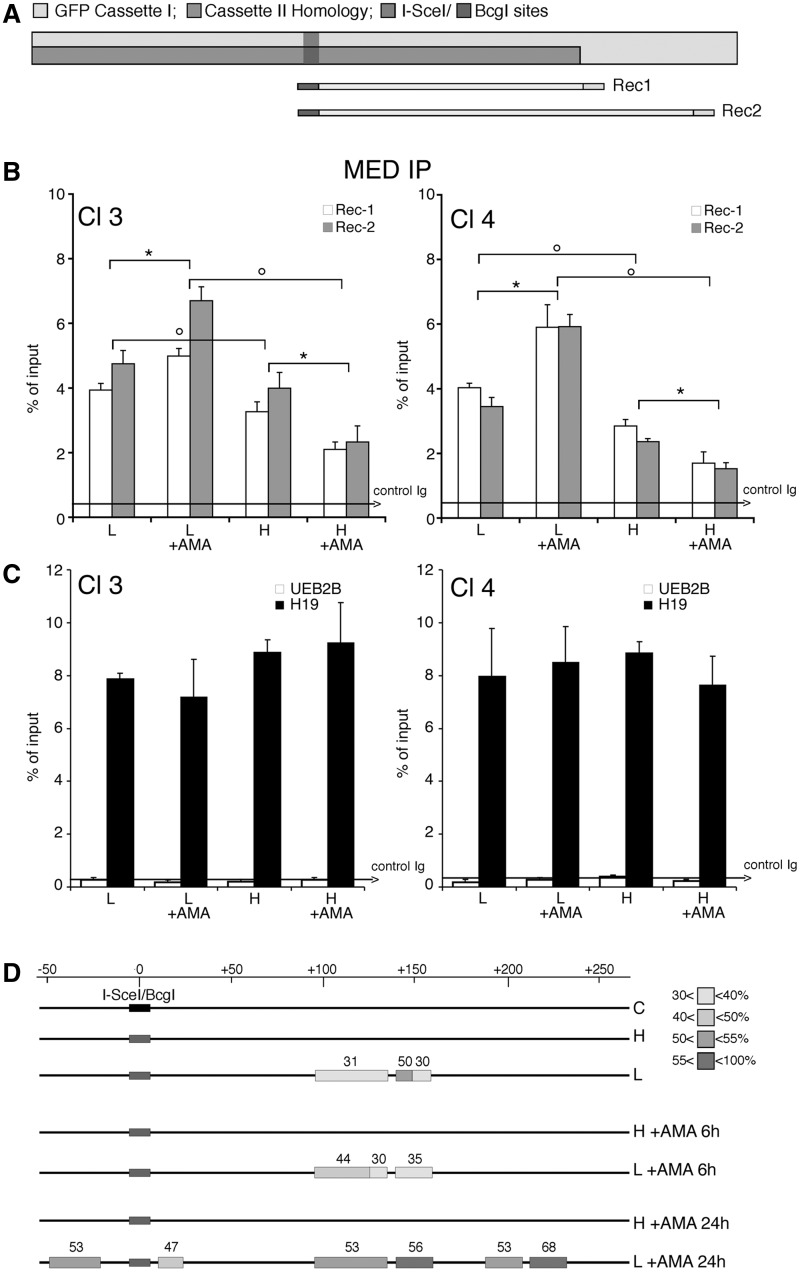
DNA methylation and chromatin modifications of the DSB region in cells exposed to α-amanitin during repair. (**A**) Location of Bcg, Rec1 and Rec2 primers, which recognize selectively recombinant GFP. Cassette I and II refer to Figure 1. (**B**) MEDIP with anti-5mC antibodies of recombinant GFP gene. Clones 3 and 4 were treated with α-amanitin for 24 h as described in Figure 2 and sorted 5 days after I-SceI as described in ‘Materials and Methods’ section. Content of 5mC is higher in L cells compared with H cells, α-amanitin also increases the levels of 5mC in L cells and lowers them in H cells. The results are similar for both amplicons (REC1 and REC2). All data derive from three independent experiments performed in triplicate (mean ± SD; n = 9). Differences between treatments were tested for statistical significance using Student’s matched pairs *t* test: *P < 0.01 as compared with the each control (α-amanitin treated versus untreated cells). Differences between cells (H versus L) were tested for statistical significance using Student’s *t* test: *P* < 0.01. (**C**) MEDIP analysis of the methylated H19 DMR (differentially methylated region) and the hypomethylated UBE2B genes in clone 3 and 4, treated with α-amanitin, as indicated in B. Longer exposure (48 h) to α-amanitin did not significantly alter the methylation pattern seen at 6 or 24 h assayed by bisulfite analysis (Supplementary Figure S4). (**D**) Bisulfite protection of GFP chromatin in L and H cells. Clone 4 cells were treated with α-amanitin for 6 or 24 h after transfection and sorted as indicated in ‘Materials and Methods’ section. Chromatin was purified as described in ‘Materials and Methods’ section, denatured and treated with sodium bisulfite. DNA was extracted, amplified, cloned in TOPO TA vector and sequenced. The amplified segment corresponds to the Rec1 region and primers were designed for the bisulfite-converted (+) strand. The boxes represent stretches of nonconverted dCs present in the GFP sequence. At least 15 independent GFP molecules were analyzed for each treatment, including cells not exposed to I-SceI (C). The numbers with the grayscale boxes represent the percentage of the molecules protected from bisulfite conversion in the regions indicated by boxes. The scale shows the coordinates of the GFP sequence relative to the DSB (indicated as 0 or *I-SceI/BcgI* site).

We therefore asked if α-amanitin altered the DNA methylation profile of the repaired GFP gene. Clones 3 and 4 were treated with α-amanitin (6–24 h), sorted 5 days later into H and L clones and analyzed by MEDIP assay with specific antibodies against 5-methylcytidine (anti-5mC) with primers indicated in Figure 3A. Figure 3B shows that anti-5mC recognizes the region 3′ to the *I-SceI* site in the repaired GFP. As predicted, the frequency of 5mC was higher in L clones than in H clones. Consistent with GFP expression profiles shown in Figure 2, α-amanitin increased the levels of 5mC in the L clones. The changes in 5mC levels were specific to the recombinant GFP segment, since the methylation status of the β-actin 5′ CpG island did not change (data not shown). Additionally, the methylation status of H19-DMR (Differentially Methylated Region), or UBE2B gene (NC_000005.9), used as positive and negative controls of MEDIP immunoprecipitation, did not change after α-amanitin (Figure 3C). To visualize directly the methylation status of the repaired segment of GFP in α-amanitin-exposed cells, we performed bisulfite analysis of the GFP gene in treated cells (Supplementary Figure S4). The repaired GFP gene just 3′ to the DSB was selectively hypermethylated or hypomethylated in L and H cells, respectively. Treatment with α-amanitin for 6 or 24 h accentuated these alterations of methylation: L clones became more methylated and H clones less methylated than untreated cells. Longer exposure (48 h) to α-amanitin did not significantly alter the methylation pattern seen at 6 or 24 h (see the legend of Supplementary Figure S4).

To explore further the local chromatin changes induced by methylation and the effects of α-amanitin on this process, we analyzed sites on the GFP gene that were protected from bisulfite conversion. Briefly, chromatin of L and H cells was fixed with formaldehyde, heat denatured and exposed to bisulfite. By probing GFP DNA, we can detect specific DNA segments protected, most likely by bound proteins, that block C to T conversion by bisulfite or by structures preventing single-strand formation (Figure 3D). The protected segment of GFP corresponds to the region containing the methylated sites at the 3′ end of *I-SceI*, as shown in Supplementary Figure S4. We found no protected areas in the H clones, whether or not they were treated with α-amanitin. Exposure to α-amanitin enhanced protection against bisulfite in most of the regions found to have increased DNA methylation after repair (compare Figures 3 and Supplementary Figure S4).

We propose that stalled RNA polymerase during repair favors the recruitment of enzymes that methylate the repaired DNA, consolidating the methylation of L clones. This event occurs only during repair because stalling Pol II before or after DSB repair does not modify GFP methylation and expression.

### Transcription modifies methylation of the repaired gene

The α-amanitin experiments suggest that the transcription machinery plays a major role in repair-induced methylation. We chose to inhibit transcription in a different fashion, by treating the cells with actinomycin-D (Act-D) for 6 h after repair. In contrast to α-amanitin, Act-D depletes RNA polymerase II from chromatin (20).

**Figure 4.**
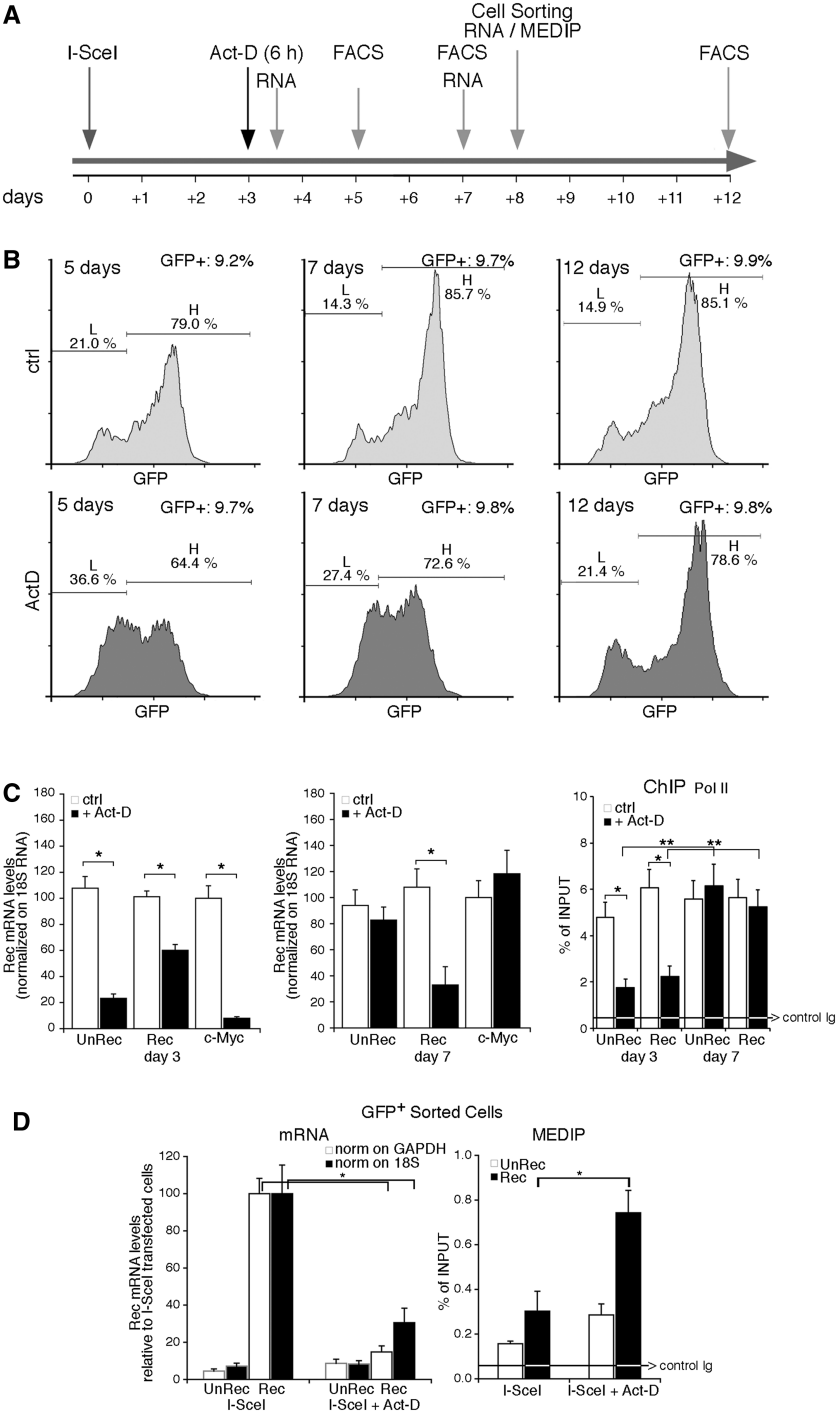
Transient exposure of recombinant cells to Actinomycin D increases methylation of the repaired gene. Panel (**A**) shows the time frame of actinomycin-D (Act-D) treatment and the assays performed. The cells were transfected with I-SceI expression vector and 72 h later were exposed to Act-D (0.05 mg/ml) for 6 h. Act-D did not induce detectable modifications of the cell cycle by PI analysis (G1 50 ± 2 versus 50 ± 3; S 23 ± 1.2 versus 25 ± 1.6; G2/M 27 ± 1.6 versus 25 ± 1.8 in the presence of 6 h Act-D); the cells were viable and RNA polymerase II was depleted from the chromatin. Five days after the treatment, the recombination frequency, measured by qPCR and GFP transcription were comparable between treated and untreated cells. The arrows indicate the time window of RNA analysis, MEDIP, FACS and cell sorting, relative to I-SceI transfection. (**B**) FACS analysis (a representative of five independent experiments) was performed as described in Figure 1 at 5, 7 and 12 days after I-SceI transfection (2, 4 and 9 days after Act-D treatment, respectively). Panel (**C**) Left. GFP mRNA accumulation assayed by qPCR after Act-D treatment (3 days after I-SceI transfection and 10 h after Act-D, or 7 days after I-SceI and 96 h after Act-D) normalized to 18 S RNA. Recombinant (Rec) and nonrecombinant (UnRec) mRNA levels are expressed as percent of untreated levels ± SD because the absolute mRNA levels cannot be compared because of the differences of the efficiency of the primers. The same results were obtained normalizing GFP RNA to GAPDH mRNA. Differences between treatments were tested for statistical significance using Student’s matched pairs *t* test: *P < 0.01 as compared with the each untreated control. Right. RNA polymerase II recruitment on recombinant and nonrecombinant GFP chromatin after Act-D treatment. ChIP with anti-Pol II large fragment antibodies of chromatin extracted from Act-D–treated cells 3 days after I-SceI transfection (10 h after Act-D) or 7 days after I-SceI (96 h after Act-D). *P < 0.01 compared with the each untreated control; **P < 0.01, 3 days compared to 7 days time point; the average of immunoprecipitated DNA with a control Ig is reported on the bar graph. (**D**) GFP mRNA levels and MEDIP assay at day 8 on sorted GFP^+^ cells. Left: Recombinant (Rec) and nonrecombinant (UnRec) primers were used to quantify GFP mRNA by qPCR and to measure the contamination of nonrecombinant GFP negative cells. The values were normalized to GAPDH (white columns) or 18 S (black columns) RNAs. Rec mRNA levels are shown as percent of the levels found in control cells (I-SceI transfected/Act-D untreated cells); UnRec mRNA levels are expressed as percent of control (untransfected DRGFP cells) (mean of three experiments in triplicate ± SD). *P < 0.01 as compared with untreated control. Right: 5mC content was carried out on sorted GFP^+^ cells (H and L) as indicated in panel A. Specifically, we analyzed the 5mC content of (i) a segment of the GFP promoter, 1 kb upstream the DSB (oligo b and c, see Supplementary Table S1); (ii) the region 3′ to the DSB, which was methylated by HDR; and (iii) H19 and UE2B genes, as controls of hypermethylated and undermethylated genes, respectively, and to monitor the efficiency of MEDIP assays. The 5mC levels in these regions, except the segment 3′ to the DSB, were not modified by 6 h Act-D treatment (data not shown). 5mC levels are expressed as percentage of input (mean ± SD of three experiments in triplicate); the average of immunoprecipitated DNA with a control Ig is reported on the bar graph. *P < 0.01 as compared with the each untreated control. Act-D, administered 27, 30 and 35 days after I-SceI for 6 h, transiently inhibited transcription, but did not change GFP gene methylation.

We were unable to use Act-D during repair, owing to inhibition of HR by the drug (data not shown). After repair, 6 h exposure to Act-D did not alter DNA replication or HDR (legend of Figure 4). Under these conditions, the treatment with Act-D prevented the accumulation of H clones at 2 and 4 days later (5 and 7 days after I-SceI transfection), although the number of GFP^+^ cells was similar in all samples (∼9.5%), and the recombination frequency was unaltered (Figure 4B and data not shown). This finding suggests that the conversion of L to H cells after repair requires transcription (Figure 4B). To confirm the effectiveness of Act-D and to explore the mechanism of inhibition of H cell formation, we measured mRNA levels of several genes. Specifically, we analyzed the accumulation of stable and unstable RNAs: (i) recombinant (Rec) and nonrecombinant (UnRec) GFP; (ii) c-Myc (0.5–1 h half-life) (21); (iii) β-actin (8–12 h half-life; data not shown) (22); and (iv) 18 S ribosomal RNA, 10 and 96 h after Act-D treatment. Figure 4C (left panel) shows the expected reduction in c-Myc, unRec and Rec mRNA levels 10 h after Act-D treatment (day 3). Rec mRNA was more stable than unRec mRNA. However, 96 h after Act-D exposure (day 7), UnRec and c-Myc mRNA concentrations returned to control values, whereas Rec mRNA levels remained lower than controls (Figure 4C, middle panel). Depletion of Pol II after Act-D exposure and the restauration of GFP-bound Pol II were confirmed by ChIP analysis of Un-Rec and Rec DNA (Figure 4C, rightmost panel). After 12–15 days, the increase of methylation and the inhibition of transcription of the GFP gene, induced by Act-D, progressively disappeared. Resumption of transcription promoted methylation loss during this period and accumulation of H cells from L cells (Figure 4B). These changes occurred only 2–3 weeks after the repair and were specific to repaired DNA because Act-D did not change the expression of undamaged GFP and, when administered 27–30 days after repair, did not modify GFP methylation (Supplementary Figure S5). We note that the time window of Act-D responsiveness (3–15 days after exposure to I-SceI) corresponds to the time required to stabilize the L/H cell ratio (Figure 1), suggesting that stabilization of the DNA–chromatin domain induced by HDR occurs in this interval. Collectively, these data indicate that after repair transcription converts a fraction of L to H cells by favoring loss of methylation.

### Hierarchical clustering analysis of GFP methylation in repaired clones links discrete methylation states to gene expression variation

The data shown above indicate that the original methylation profiles induced by HDR are remodeled in a transcription-dependent fashion during the first 15 days after repair. The pattern eventually stabilizes, locking the epigenetic status of the repaired DNA in each cell (see Supplementary Movie, cycles I and II/III). By using hierarchical clustering analysis of bisulfite-treated GFP molecules before and after HDR, we were able to track and identify the original methylation profiles (epialleles) induced by HDR and modified during transcription. We also were able to link the methylation states of epialleles to GFP expression levels, since the bisulfite analysis was carried out on fluorescent-sorted cells. Clones expressing intermediate levels of GFP (L2 and H2) contain a set of GFP epialleles originating from a common GFP precursor segregating in the L fraction. This epiallele precursor in L cells generates many similar epialleles as a result of losing methyl groups (Supplementary Figures S6 and S7). These sites are shared by L2 and H2 clones and are located in 2 symmetric domains downstream of the DSB, spanning the length of a nucleosome (150 bp) (Supplementary Figure S7C and D). The sites are demethylated by 5-AzadC and methylated by Act-D treatments (Supplementary Figure S7C and D or data not shown). These data definitely link gene expression to specific methylation states and explain the stochastic expression of GFP after HDR (see Supplementary Movie).

### DNMT3a is transiently recruited to repaired GFP and stimulates DNA methylation

**Figure 5.**
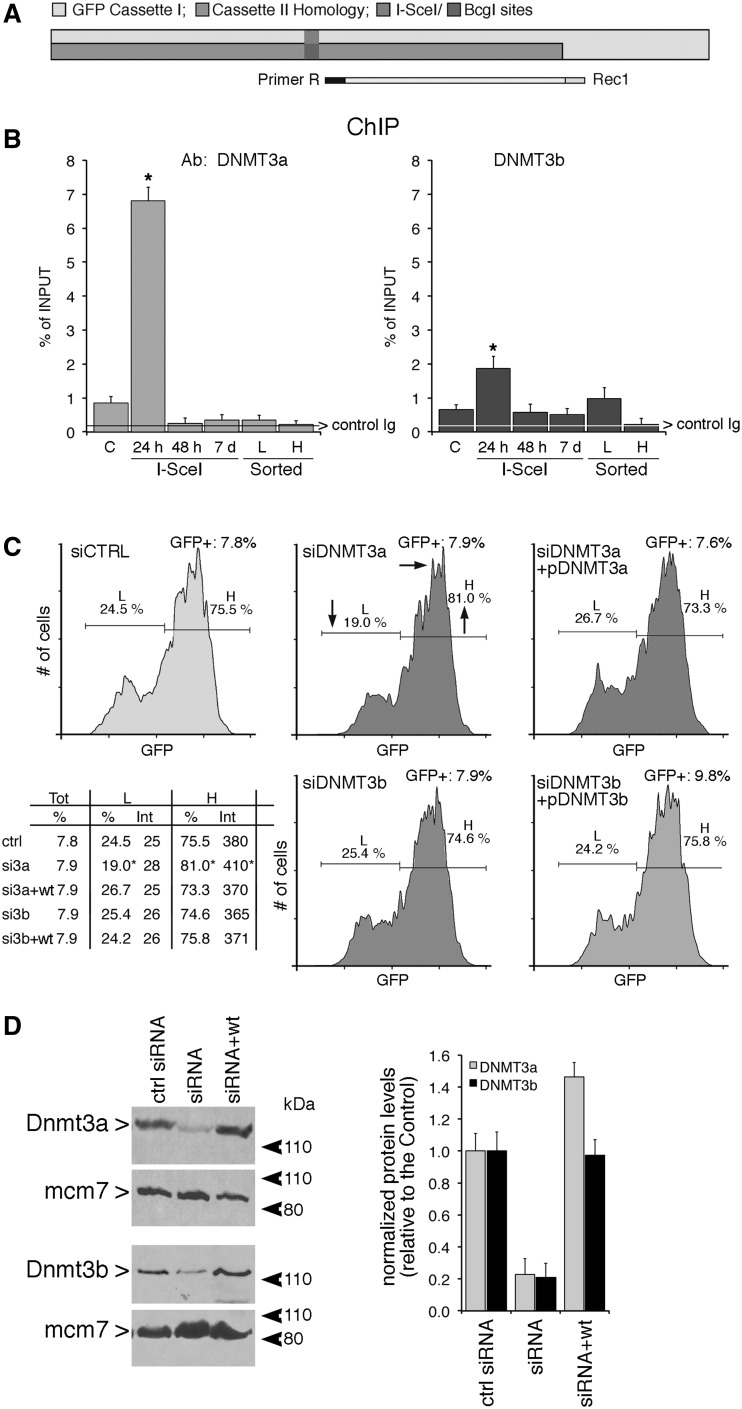
DNMT3a and 3b are recruited to the DSB early during repair, but only DNMT3a is necessary for generation of L cells (**A** and **B**) Recruitment of DNMT3a, DNMT3b to the *I-SceI* chromatin. Cells were transfected with I-SceI and 24 h, 48 h or 7 days later, were fixed, collected, chromatin-extracted and subjected to ChIP analysis with specific anti-DNMT3a and DNMT3b antibodies. The specific primers used to amplify the GFP cassette I are indicated in (A). Data represent the fraction of immunoprecipitated DNA relative to the input chromatin-DNA present in the reactions (% of input; mean ± SD; *n* ≥ 9); the average of immunoprecipitated DNA with a control Ig is reported on each bar graph. *P < 0.01, paired *t* test. (**C**) Silencing the expression of DNMT3a reduces L cells. Cells were electroporated with the siRNA targeting DNMT3a and DNMT3b (see ‘Materials and Methods’ section and protocol S1) and analyzed 7 days later, when L and H cells were clearly separated. On the bottom left panel, statistical analysis derived from three independent experiments is shown. *P < 0.01, paired *t*-test comparing GFP intensity, Chi Square (χ2) comparing the percentage of L/H cells. The horizontal and vertical arrows in the central inset indicate the shift in fluorescence intensity and in the distribution of L and H cells, respectively. Treatment with 5azadC (10 µM for 2 days, 48 h after I-SceI transfection) rescued completely the loss of L cells (intensity and % GFP^+^ cells) induced by DNMT3a overexpression in siDNMT3a-silenced cells (data not shown). (**D**) Western blot analysis of DNMT3a and 3 b in silenced cells. Total cell extracts were prepared 48 h after electroporation and analyzed by immunoblot with the specific antibodies indicated. On the right is shown quantitative analysis derived from three immunoblots (mean ± SD).

We previously reported that the hypermethylated L cell population was not found in a mutant lacking the maintenance DNMT1. In contrast, hypermethylation of the repaired gene was seen in both DNMT3a^−^^/−^ and DNMT3b^−^^/−^ mutants (9). However, loss of methylation induced by repair in stable DNMT1 mutant cells may be the indirect consequence of lack of propagation of methylation in daughter cells by DNMT1. Since large stretches of DNA are resynthesized during homologous recombination and are devoid of methylation marks, it is possible that de novo DNMTs such as DNMT3a and 3 b have a role during or early after repair, and that DNMT1 may propagate the methylation marks set by DNMT3a and/or 3 b during replication. To investigate this possibility, we analyzed the recruitment of DNMT3a and 3 b to the I-SceI–cleaved chromatin. Figure 5A and B show that both DNMT3a and DNMT3b were recruited to the *I-SceI* site 24 h after the onset of DSB formation and rapidly disappeared (48 h). We then selectively silenced DNMT3a and 3 b during repair and analyzed the distribution of L and H cells. Figure 5C shows that the yield of L cells was significantly reduced and both the number and GFP fluorescence intensity of H cells increased when DNMT3a expression was silenced. In contrast, depletion of DNMT3b did not alter the ratio of L and H cells (Figure 5C). Expression of wild-type enzyme in DNMT3a-silenced cells prevented the loss of L cells. The changes of GFP expression levels were caused by DNA methylation, since the rescue of L cells by DNMT3a was prevented by treatment with 5azadC (Figure 5C).

In conclusion, we propose that DNMT3a helps the formation of hypermethylated clones and DNMT1 propagates these methylation patterns through at least several generations. This finding reinforces the notion that maintenance and de novo methyl transferases cooperate (23).

### Np95 is recruited to repaired GFP and stimulates DNA methylation

**Figure 6.**
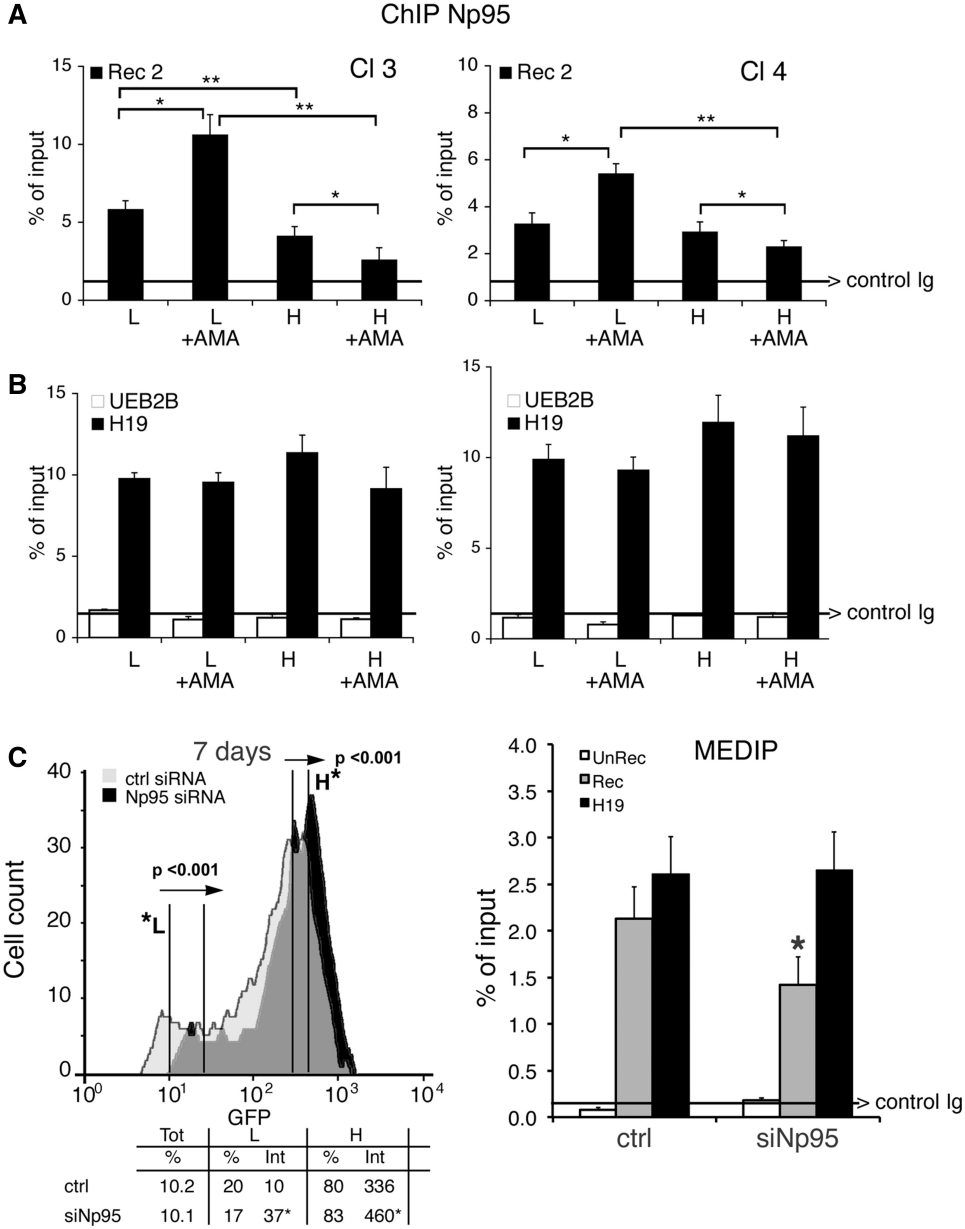
Np95 (UHRF1) is recruited to repaired GFP and stimulates DNA methylation. (**A**) ChIP with anti-Np95 antibodies of sorted cells exposed to α-amanitin during repair. Clones 3 and 4 were transfected with I-SceI and treated with α-amanitin for 24 h as described in Figure 2. The cells were sorted 5 days after I-SceI transfection and chromatin was collected from formaldehyde-fixed cells and subjected to ChIP analysis with specific antibodies to Np95. Primers Bcg and Rec2 were used to amplify recombinant GFP DNA. The data derive from three independent experiments performed in triplicate (mean ± SD; n = 9). Differences between treatments were tested for statistical significance using Student’s matched pair *t*-test: *P < 0.01 as compared with the each control (α-amanitin treated versus untreated cells). Differences between cells (H versus L) were tested for statistical significance using Student’s *t*-test: **P < 0.01. (**B**) ChIP analysis of Np95 on H19 DMR and UBE2B genes. qPCR was carried out with specific H19 DMR and UBE2B primers on the same samples indicated above. The fraction of immunoprecipitated DNA by control Ig is reported on each bar graph. (**C**) DRGFP cells (pool of clones; clone 3 and 4 are not shown here) were transiently transfected with a mixture of siRNAs targeting specifically human NP95 or control scrambled siRNA (ctrl) and the mouse I-SceI expression vector (see ‘Materials and Methods’ section). Six days later, the cells were subjected to FACS analysis and MEDIP. The left panel shows a representative experiment: arrows indicate the shift in silenced cells of GFP fluorescence intensity. The columns below the fluorescence plot show (i) the number of GFP^+^ cells (Tot, expressed as percentage of cells); (ii) the mean fluorescence intensity (Int.); and (iii) Percentage of L and H cells on GFP^+^ cells. Mean fluorescence intensity at day 7 increased from 10 to 37 in L cells and from 336 to 460 in H cells (left panel). FACS analysis was performed in triplicate in at least three experiments. Differences between treatments were tested for statistical significance using Student’s matched pair *t*-test: *P < 0.001 as compared with the each control (siRNA-treated versus untreated cells). Samples expressing NP95 wild-type and control cells were treated with 1 µM 5azadC for 1 day (48 h after I-SceI), and the differences in fluorescence intensity was used to quantify methylation-dependent changes of GFP expression. The panel on the right shows the results of MEDIP immunoprecipitation with anti-5mC antibodies in control and siRNA-treated samples. Np95 depletion by siRNA did not modify the methylation status of stably methylated genes, such as H19 (DMR) and β-actin CpG island. *P < 0.01 for *t*-value (matched pair test) relative to the cells treated with control scramble siRNA (CTRL). Data are expressed as mean ± SD, *n* = 9; the average of immunoprecipitated DNA with a control Ig is reported on the bar graph.

We reported that DNMT1 was required for hypermethylation of repaired GFP. We now ask if proteins that modify DNMT1 activity influence DNA methylation at the repaired DSB. We probed for Np95 (also known as UHRF1 or ICBP90), a protein that binds to DNMT1, DNMT3a, DNMT3b and PCNA and stimulates methylation of hemi-methylated DNA (24–26). ChIP analysis of GFP chromatin from clones 3 and 4 showed that Np95 preferentially accumulated on the repaired chromatin of the L clones. Treatment with α-amanitin during repair significantly amplified or decreased Np95 recruitment to GFP chromatin in L or H cells, respectively (Figure 6A). Note that the binding of Np95 to H19, UEB2B or β-actin CpG island chromatin was unaffected by α-amanitin (Figure 6B and data not shown). Thus, the association of Np95 with the DSB of GFP DNA appears to be linked to hypermethylation and reduced GFP expression in the L cell population.

To test whether Np95 recruitment to recombinant chromatin was relevant to repair-induced methylation, we selectively silenced Np95 expression during recombination. We measured GFP expression, DNA methylation in the repaired segment and the frequency of recombination. Figures 6C (left panel) and Supplementary Figure S8 show that silencing of Np95 expression significantly enhanced fluorescence intensity in both the L and H cell fractions. Np95 depletion did not affect recombination frequency (Supplementary Figure S8A) but induced loss of methylation at the 3′ end of the repaired GFP gene (Figure 6C, right panel). Under the same conditions, Np95 depletion did not modify the methylation status of β-actin CpG island, or stably methylated gene, H19 (DMR) (see the legend of Figure 6C). Overexpression of mouse wild-type Np95 reversed the effects of the silencing and reduced GFP expression (Supplementary Figure S8B).

Np95 interacts with several proteins involved in chromatin remodeling, specifically those that set repressive marks on histones, such as SUV39 and EZH2 (27,28). Indeed, 24 h after DSB induction, the I-SceI chromatin shows an accumulation of histone repressive (H3K9 m2-m3) and a reduction of positive H3K4 (m2 and m3) marks, respectively [(13) and data not shown]. To test if SUV39 and EZH2, which also interact with DNMT1 (27,28), play a role on DNA methylation induced by damage and repair, we silenced their expression during repair and determined the distribution of L and H cells. Knockdown of these proteins did not significantly modify the intensity of the GFP signal in either L or H cells (Supplementary Figure S9A). Although a modest decrease in GFP expression in SUV39-depleted cells was caused by inhibition of recombination (Supplementary Figure S9C), the levels of GFP methylation were not modified in cells in which SUV39 and EZH2 were silenced (Supplementary Figure S9D).

### GADD45a binds DSB and inhibits de novo methylation induced by HDR

To identify a DNMT1 partner that inhibits DNA methylation during repair and generates H cells, we monitored GADD45a (G45a) expression and localization after DSB formation. We recently found that GADD45A binds hemi-methylated DNA, inhibits DNMT1 *in vitro* and *in vivo* and reduces the fraction of L cells (18), suggesting that GADD45A promotes loss of methylation on the repaired DNA (29,30).

**Figure 7.**
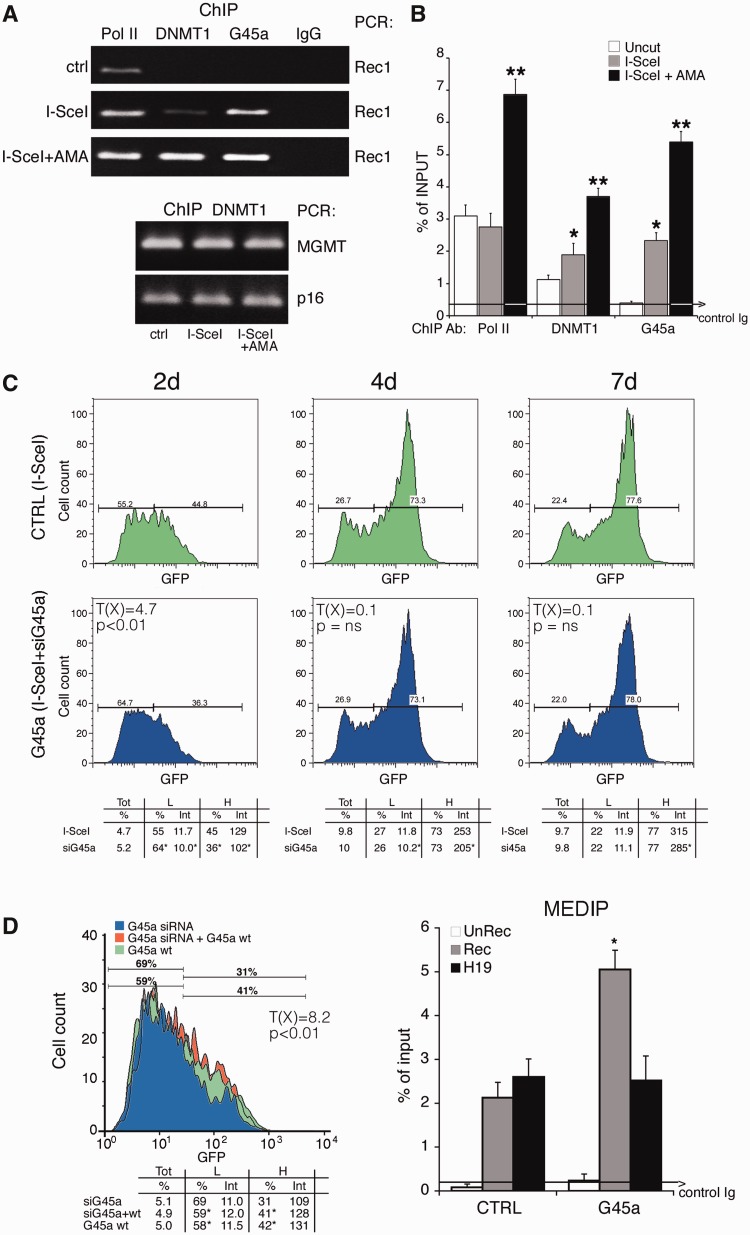
GADD45 is recruited to the DSB and transiently inhibits de novo methylation induced by HDR. (**A**) ChIP analysis with anti-GADD45A, DNMT1 and RNA polymerase II large fragment antibodies in HeLa cells, transfected (36 h) with I-SceI. Twelve hours after transfection, an aliquot of cells was treated for 24 h with α-amanitin and processed as described in ‘Materials and Methods’ section. Bcg and Rec1 primers were used for semiquantitative PCR. Two methylated genes, MGM and p16, were used as controls for DNMT1 ChIP. Control IgG represents an average of nonimmune immunoglobulins used in ChIP. (**B**) Quantitative analysis by qPCR of at least three ChIP experiments in triplicate (*n* ≥ 9). Differences between treatments were tested for statistical significance using Student’s matched pairs *t*-test: *P < 0.01 as compared with uncleaved control; **P < 0.01 compared with I-SceI. The average of immunoprecipitated DNA with a nonimmune Ig is reported on the bar graph. (**C**) DRGFP cells (pool of clones) were transiently transfected with siRNA pools targeting specifically GADD45A or control scrambled siRNA (ctrl) and the I-SceI expression vector (see ‘Materials and Methods’ section). After 2, 4 and 7 days, the cells were subjected to FACS analysis as described in Figure 1. FACS analysis was performed in triplicate in at least three experiments. Differences in GFP expression between control and GADD45A-silenced cells were tested for statistical significance using the Chi Square test, T(X) (Population Comparison module of the FlowJo software from Tree Star). Differences of L and H (percentage and intensity) were tested for statistical significance using Student’s *t* test: *P < 0.01 (see Supplementary Figure S10). χ2 value (4.7), 2 days after I-SceI (control and GADD45A-silenced cells) (*P* < 0.01); at day 4 and 7, χ2 value was not discriminant as day 2, although differences in fluorescence intensity of L an H cells between the control and GADD45A-silenced cells were significant (*P* < 0.02). All samples were treated with 1 µM 5azadC for 1 day (48 h after I-SceI) to quantify methylation-dependent changes. (**D**) Left panel. Forced expression of GADD45A increases GFP expression. Cells were exposed to siRNA targeting the 3′ UTR human GADD45A alone or in combination with vector expressing GADD45A. GFP fluorescence and Rec mRNA were analyzed 4 days later. The levels of specific GADD45A mRNA, the frequency of recombination in GADD45A-depleted cells and the statistical analysis of GFP expression are shown in Supplementary Figure S8. Differences between populations (control and GADD45A-silenced cells) were tested for statistical significance using the Chi Square test (Population Comparison module of the FlowJo software). Cells expressing CMV-EGFP–treated with etoposide or transfected with Ga45a expressing vector did not change GFP expression. Right panel. 5mC content of recombinant GFP in cells silenced for GADD45A. Four days after transfection, the cells were subjected to MEDIP assay. GADD45A depletion by siRNA did not modify the methylation status of stably methylated genes, such as H19 (DMR) and β-actin CpG island. *P < 0.01 for *t* value (matched pair test) relative to cells treated with control scramble siRNA (CTRL). All the samples in independent experiments were treated with 1 µM 5azadC for 1 day (48 h after I-SceI) to quantify methylation-dependent changes. The average of immunoprecipitated DNA with a control Ig is reported.

We first measured GADD45A mRNA levels in cells exposed to I-SceI or to the DNA-damaging agent, etoposide. GADD45A mRNA was induced by I-SceI and decreased to pre-induced levels 48 h after I-SceI transfection (Supplementary Figure S10). We next asked if GADD45A accumulated on DNA during HDR. ChIP analysis shows that GADD45A was recruited to GFP chromatin 48 h after I-SceI expression, confirming a previous observation (18). Recruitment of GADD45A, as well as DNMT1 and Pol II, was further stimulated by α-amanitin (Figure 7A and B). Note that DNMT1 accumulation on MGMT and p16, genes normally methylated in Hela cells, was not stimulated by I-SceI expression or α-amanitin (Figure 7A, lower panel).

We next tested the effects of silencing GADD45A on recombinant DNA methylation. Figure 6C shows that GADD45A knockdown (Supplementary Figure S11) inhibited GFP expression at 2 and 4 days after the damage. However, although reproducible, this effect, which was not noted previously (18), was transient; it was statistically significant at day 2 and progressively disappeared at 4 and 7 days after I-SceI expression (Figure 7C and Supplementary Figure S11 panels A and C). The consequences on GFP expression of GADD45A silencing at 2 days were reversed by co-transfection with a mouse GADD45A expression vector (Figure 7D, left panel). GADD45A silencing did not alter the frequency of recombination (Supplementary Figure S11D) but methylation of GFP was significantly stimulated, as shown by MEDIP analysis (Figure 7D, right panel). Under the same conditions, GADD45A depletion did not modify the methylation status of β-actin CpG island or of stably methylated genes, such as H19 (DMR) (Figure 7D).

The transient effects of GADD45A depletion on GFP expression may be dependent on the transient rise of the protein (18) and mRNA levels during damage and repair (Supplementary Figure S10). To address this issue, we overexpressed the wild-type protein, 2 days after I-SceI transfection, when endogenous protein levels were already low. Under these conditions, Ga45a stimulated GFP fluorescence intensity in H cells for longer periods (4–7 days after I-SceI), but at day 10 from the DSB, the effects disappeared (Supplementary Figure S11E and data not shown). However, 1 month after the DSB or in cells expressing CMV-EGFP, forced expression or induction of GADD45A by etoposide did not modify GFP levels (see the legend of Figure 7).

Taken together, these results indicate that Np95 and GADD45A favor the generation of L and H cells, respectively, during HDR.

## DISCUSSION

### Mechanism of DNA repair-induced methylation

**Figure 8.**
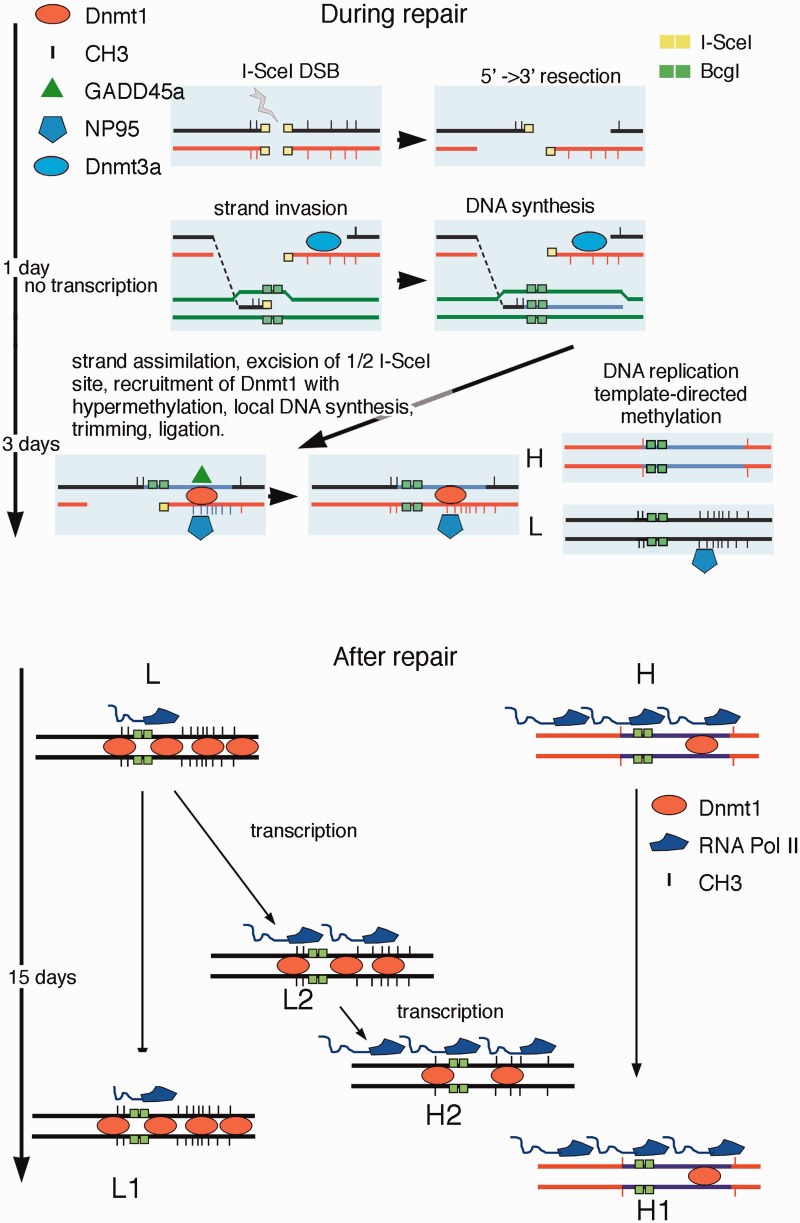
Targeted methylation during and after homologous repair. The cartoon represents a schematic model illustrating the events during and after repair. The DSB undergoes 5′ –> 3′ end resection and one of the 3′ free single strand end invades the DNA of the GFP cassette II. The half *I-SceI* site is removed (flap removal) and new DNA is synthesized. Eventually, the invading strand returns to the original configuration and directs the synthesis of new DNA at the *BcgI* site corresponding to the DSB, according to the SDSA model (Synthesis Directed Strand Annealing) (31). We propose that the asymmetric distribution of methylated CpGs in repaired GFP is caused by selective invasion of the (+) strand. The (−) strand, blocked by stalled RNA Pol II (DNMT1 and 3 a), becomes a preferential target of DNMT1-Np95. The hemi-methylated DNA is replicated and generates H and L cells. After repair, transcription resumes and RNA Pol II-DNMT1 is associated with methylation/demethylation cycles (32) that in 15 days may remove some methyl groups in a subpopulation of L cells, leading to the conversion of L2 to H 2 cells.

The results shown here argue for a link between HDR and DNA methylation at the site of a repaired DSB. Without DNA damage and repair, the expression of GFP is stable and uniform (Supplementary Figure S6, the red peak). DSB formation within GFP and repair by HDR significantly alter the methylation pattern of GFP in two steps. We propose that some actors at this phase are DNMT1/3 a, Np95 and GADD45A, which transiently maintain the processed DSB 3′ segment hemi-methylated, until replication generates methylated and hypomethylated daughter molecules. Figure 8 shows a simplified scheme describing the main events during and after DSB repair: (i) DNMT1 and DNMT3a are recruited to the DSB with Ga45a and NP95. DNMT3a is recruited in the first 24 h after damage and transiently cooperates with DNMT1 to methylate repaired DNA. At 48 h, Np95 and Ga45a amplify or limit transiently, respectively, DNMT1 activity on the hemi-methylated DNA, until replication duplicates the methylated and unmethylated DNA strands. This is better shown in the video presented in the Supplementary Movie, in which time lapse microscopy offers a unique snapshot into homologous repair. The appareance of the GFP signal in I-SceI synchronized cells can be monitored in the first and second cycle after recombination, relative to the GFP signal, generated by HDR. In the first cycle, H and L cells are formed from the same cell (square in the Supplementary Movie); in subsequent cycles, H and L cells stably propagate in culture the H or L phenotype (circle in the Supplementary Movie); (ii) After repair, transcription resumes at day 2–3 after DSB and progressively modifies local methylation profiles until the local domains of the *I-SceI* chromatin (loop A in H cells and loop C in L cells) are stabilize. We believe that this strand-selection mechanism accounts for the ∼1:1 L/H ratio early after repair (Figures. 1 and 4). In fact, GADD45A exerts its action early during repair (2–4 days), when the L/H ratio is close to 1 and before significant remodeling of methylation occurs (Figures 1, 4 and 7). Stalled RNA Pol II by α-amanitin during repair may facilitate targeting DNMT1/3 a complex to the 3′ end (−) transcribed strand, thus promoting hyper-methylation of the 300 bp repaired DNA segment that lies 3′ to the DSB relative to transcription orientation (Figure 3D and Supplementary Figure S4). The 3′ end (+) strand, free from transcription proteins, probably is more prone to invade and find the homologous region to direct the annealing, the synthesis and ultimately the repair of the DSB (Synthesis Directed Strand Annealing, SDSA) (31). This mechanism may account for the relatively high efficient HDR in our system.

### Remodeling of methylation by transcription after repair

The second step of methylation induced by HDR begins ∼48 h after formation of the DSB. At this point, repair is terminated, but chromatin and DNA continue to undergo epigenetic changes (9,13). H cells progressively increase and are similar in terms of methylation profile to a subpopulation of L cells (L2 in Supplementary Figure S6). Accumulation of these L2/H cells is favored by continuous transcription of GFP because transient inhibition of transcription after repair shifts the L/H ratio and favors accumulation of methylated clones (L2 in Figure 4D). We obtained essentially the same results shown in Figure 4 by transiently blocking transcription after repair with a dominant negative cdk9-expression vector, which inhibits phosphorylation of elongating RNA polymerase II (G.R., unpublished observations). However, 27, 30 and 35 days after DNA damage, inhibition of transcription by exposure to Act-D or expression of the dominant negative cdk9 did not alter methylation or expression of GFP (Supplementary Figure S5). These data indicate that inhibition of transcription per se does not trigger de novo methylation (33–35) and suggest that transcription may favor active demethylation. In fact, depletion of base excision repair (BER) enzymes (OGG1; APE1) or TDG increased methylation of repaired GFP similarly to Act-D treatment (data not shown), in agreement with the notion that transcription is associated with DNA methylation-demethylation (32,36) and DNA oxidation cycles (37). We note that the different effects of α-amanitin and Act-D are related to the ability of these drugs to increase (α-amanitin, Supplementary Figure S2B) or deplete (Figure 4D) RNA polymerase II from chromatin: (i) Stalled pol II during repair increases targeting and recruitment of DNMT1-Np95 on the DSB and favors accumulation of L clones; (ii) depletion or loss of pol II by slow resolution of Act-D/DNA inhibit transcription and active demethylation.

We suggest that transcription of damaged-repaired DNA is associated with stochastic replacement of methylated C by BER or nucleotide excision repair (NER) followed by repair synthesis (38,39). The events in this phase are distinct from those leading to the generation of H and L cells during repair, which are amplified by stalled RNA polymerase II and are dependent on Np95 and Ga45a. Under our conditions, GADD45A, transiently induced by DSB, recruited to the DSB, enhanced accumulation of hypomethylated clones (H) by inhibiting DNMT1 [Figure 7, Supplementary Figure S11 and (18)] and disappeared in 3–4 days. This inhibition may represent a barrier to spreading of repair-induced methylation. The opposing role of Np95 and GADD45A on DNMT1 activity is not new because DNMT1 stimulation and inhibition by Np95 and Ga45a, respectively, are required to maintain progenitor function in self-renewing somatic tissue (40).

### Evolution and stability of epialleles: qualitative analysis of methylation

Our data show that the repaired DSB in the GFP gene is marked locally by de novo methylation. Unlike the GFP system, in which we induced a site-specific DSB, DSBs in genomic DNA are essentially random in terms of sequence specificity, although the overall distribution is nonrandom, due to chromatin organization (41). Assuming that methylation marks these DSBs after homologous repair, the overall distribution of methylated sites in genomic DNA will appear random in the absence of selective pressure. We have extended our analysis to homologous targeting of GFP in ES cells and we find that genetically identical clones express variable GFP levels, due to de novo methylation or targeted gene (data not shown).

In our system, qualitative analysis of the methylation profiles, i.e. the location of methylated CpG in the various GFP molecules, 3′ end to the DSB, is able to distinguish repaired GFP molecules from nonrecombinant or uncleaved molecules (Supplementary Figures S6 and S7). This discrimination is based on the relatedness of methylation profiles, not on the total methyl CpG content. GFP DNA molecules, shown in Supplementary Figures S4 and S6, can be considered epigenetic alleles because their methylation profiles are stable and are inherited in human and mouse cells over several generations. We have applied the same type of analysis shown in Supplementary Figures S6 and S7 to several somatically methylated genes and we find that the epialleles are stable, evolve rapidly following DNA damage and can be individually tracked in a complex mixtures of cells. HDR-induced specific methylation states may be ultimately responsible for stochastic gene expression in populations of mammalian cells.

In conclusion, we propose that DNA methylation represents a damage-repair code that modifies the expression of genes in cell populations and drives adaptation to environmental challenges. Selection of methylated alleles in each cell may be relevant for the rapid evolution of cancer cell phenotypes.

## SUPPLEMENTARY DATA

Supplementary Data are available at NAR Online, including [42].

## FUNDING

AIRC IG [11364 to V.E.A.]; Epigenomics Flagship Project—EPIGEN (to C.N.R.) and Fondazione Medicina Molecolare e Terapia Cellulare, Universita' Politecnica delle Marche. Funding for open access charge: Epigenomics Flagship Project—EPIGEN (to C.N.R.).

*Conflict of interest statement*. None declared.

## Supplementary Material

Supplementary Data
