# Supplementary material for: Targeted DNA methylation by homology-directed repair in mammalian cells. Transcription reshapes methylation on the repaired gene
Source: Nucleic Acids Res. 2013 Oct 9;42(2):804–21. doi: 10.1093/nar/gkt920 (PMC3902918; doi:10.1093/nar/gkt920)
Supplement: Supplementary Data [file supp_42_2_804__index.html]

Targeted DNA methylation by homology-directed repair in mammalian cells. Transcription reshapes methylation on the repaired gene — Targeted DNA methylation by homology-directed repair in mammalian cells. Transcription reshapes methylation on the repaired gene — Supplementary Data 

# Targeted DNA methylation by homology-directed repair in mammalian cells. Transcription reshapes methylation on the repaired gene

## Supplementary Data

files

**Files in this Data Supplement:**

- Supplementary Data - pdf file
- Supplementary Data - mov file
- Supplementary Data - m4v file
