# Supplementary material for: Targeted DNA methylation by homology-directed repair in mammalian cells. Transcription reshapes methylation on the repaired gene
Source: Nucleic Acids Res. 2013 Oct 9;42(2):804–21. doi: 10.1093/nar/gkt920 (PMC3902918; doi:10.1093/nar/gkt920)
Supplement: Supplementary Data [file supp_gkt920_nar-01832-d-2013-File010.pdf]

Supplementary data for

Targeted DNA methylation by homology-directed repair in mammalian cells. Transcription reshapes methylation on the repaired gene

Annalisa Morano[1,7], Tiziana Angrisano[1], Giusi Russo[1], Rosaria Landi[1], Antonio Pezone[1], Silvia Bartollino[2], Candida Zuchegna[3], Federica Babbio[4], Ian Marc Bonapace[4], Brittany Allen[5], Mark T. Muller[5], Lorenzo Chiariotti[1], Max E. Gottesman[6], Antonio Porcellini[3*] and Enrico V. Avvedimento[1*]

1. Dipartimento di Medicina Molecolare e Biotecnologie mediche, Istituto di Endocrinologia ed Oncologia Sperimentale del C.N.R., Università Federico II, 80131 Napoli, Italy

2. Dipartimento di Medicina e di Scienze della Salute, Università del Molise, Campobasso, Itay

3. Dipartimento di Biologia, Università Federico II, 80126 Napoli, Italy

4. Dipartimento di Biologia Strutturale e Funzionale, Università dell'Insubria, Varese 21100, Italy

5. Department of Molecular Biology and Microbiology and Biomolecular Science Center, University of Central Florida, 12722 Research Parkway, Orlando, Florida 32826

6. Institute of Cancer Research, Columbia University Medical Center, New York, New York, USA

7. IRCCS CROB, via Padre Pio, 1 85028 Rionero in Vulture Italy

Supplement Index:

Supplementary Methods and References page 02

Summary of Supplementary Tables and Figures page 03

Supplementary Table S1 page 04

Legend to Supplementary Movie page 05

Supplementary Figures S1-12 pages 06-19

Supplementary Methods

Vectors and Reagents

pDR-GFP [9] plasmid was 14,735 bp containing (Supplementary Figure S12): CMV IE enhancer (1–385); chicken beta actin promoter (386-751); chicken beta-actin first intron (752-1,622); rabbit beta-globin second intron (1,623-1,670); rabbit beta-globin third exon (1,671-1,724); EGFP with a NLS (1,761- 1815) and a STOP [1] codon at I-SceI site (1,740-2,756, I-SceI at 2,135); SV40 splice/polyadenylation signal (2,757-3,023); polyadenylation signal from phosphoglycerate kinase gene (3,607-3,025) for the puromycin resistance gene (4,260-3,661); the promoter from mouse phosphoglycerate kinase gene (4,270-4,780); a truncated EGFP gene sequence (5,609-6,138); and 6,450 bp of mouse genome.

The expression vectors for GA45a and for hUHRF1(Np95) were previously described by *Lee et al 2012* [2] and *Citterio et al, 2004* [3] respectively. The expression vectors for DNMT3a and DMNT3b were from Addgene: pDNMT3a, cat.# 25328; pDNMT3b, cat.# 25163.

The siRNAs were from:

Dharmacon (ON-TARGET plus SMART pool)si-hUHRF1, cat.# L-006977; si-hEZH2, cat.# L-004218; si-hSuv39H1, cat.# L-009604; si-hGADD45, cat.# L-003893; si-Control: cat.# D-001810.

Santa Cruz Biotechnology: siDNMT3a, cat.# sc-37757; siDNMT3b, cat.# sc-37759;.

Time Lapse Microscopy of HDR.

The live imaging was done by inducing cells with Doxycycline for 24 hours and then capturing images every 5 minutes. This particular video covers a span of 30 hours at a 10x magnification. The images were taken with multiple Z-slices through the planes of the cell. Z-slices were merged into one image so that any difference in intensity that are seen cannot be attributed to differences in Z-plane positions of the cells. Cells were plated in MatTek glass bottom microwell dishes with No. 1.5 coverglass. Data was obtained using a Perkin Elmer UltraVIEW VoX confocal imaging system with a Zeiss Axio Observer Z1 inverted microscope.

Buffers Formulation.

Bisulfite DNA preparation.

Buffer A: 10 mM Tris-HCl, pH 8, 10 mM EDTA, pH 8, 0.5 mM EGTA, pH 8, 0.25% Triton X-100.

Buffer B: 0.2 M NaCl, 10 mM Tris-HCl, pH 8, 1 mM EDTA, 0.5 mM EGTA.

Buffer C: 0.3 M NaCl, 40 mM Tris-HCl, 4 mM EDTA, 1% Triton X-100.

Supplementary references

1 Pierce,A.J., Johnson,R.D., Thompson,L.H., Jasin,M. (1999) XRCC3 promotes homology-directed repair of DNA damage in mammalian cells. Genes Dev 13,2633–2638.

2 Gong XQ, Nedialkov YA, and Burton ZF (2004) Alpha-amanitin blocks translocation by human RNA polymerase II. J Biol Chem 279: 27422-27427.

3 Citterio,E., Papait,R., Nicassio,F., Vecchi,M., Gomiero,P., Mantovani,R., Di Fiore,P.P. and Bonapace,I.M. (2004) Np95 is a histone-binding protein endowed with ubiquitin ligase activity. Mol Cell Biol 24,2526-2535.

**Table S1. Complete list of DNA oligonucleotides used for PCR**.
**Figure S1. Recombination and I-SceI levels**.
**Figure S2. Exposure of cells to a-amanitin during repair increases RNA polymerase II molecules on recombinant chromatin and does not alter the rate of homologous repair.**
**Figure S3. Exposure to a-amanitin during repair, not before or after, amplifies and consolidates L and H clones.**
**Figure S4. DNA Methylation of the GFP repaired segment in a-amanitin-exposed cells.**
**Figure S5. Act-D effects on GFP expression occur only in a definite time window after after repair. 5azadC eliminates Act-D effects on GFP expression.**
**Figure S6. Hierarchical cluster analysis of methylated GFP DNA molecules after homologous repair in ES and Hela cells.**
**Figure S7. Selective methylation at the 3' of the DSB discriminates recombinant versus non-recombinant GFP molecules.**
**Figure S8. Frequency of recombination and Np95 expression in Np95-silenced cells.**
**Figure S9. Expression analysis, frequency of recombination, and GFP methylation in EZH2 or SUV39-silenced cells.**
**Figure S10. GADD45a is transiently induced by DNA damage.**
**Figure S11. GFP expression in GA45a-silenced cells.**
**Figure S12. GFP primers used to analyze recombination, methylation and DNA chromatin domains.**

Table 1S

	PRIMERS	Locus
R	5'-CCACCGGCAAGCTGCCCGTGCC-3'	pDR-GFP
Rec1	5'-TGCACGCTGCCGTCCTCG-3'	pDR-GFP
Rec2	5'-CGGCGGCGGTCACGAACTC-3'	pDR-GFP
Primer I	5'-GCTGATCTCGTTCTTCAGGC-3'	pDR-GFP
Primer L	5'-GGTACTCTGTTCTCACCCTTC-3'	pDR-GFP
Primer M	5'-GAAAGCGAAGGAGCAAAGCTG-3'	pDR-GFP
Primer I-SceI	5'-GCTAGGGATAACAGGGTAAT-3'	pDR-GFP
Primer Bcg	5'-GAGGGCGAGGGCGATGCC-3'	pDR-GFP
Bisulfite E01F	5'-GTGTGATTGGTGGTTTTAGAGT-3'	pDR-GFP
Bisulfite E02R	5'-CCATCCTCAATATTATAACAAAT-3'	pDR-GFP
Bisulfite E2F	5'-GGAGTTGTTTATTGGGGTGGTGTTTATTTTGGT-3'	pDR-GFP
Bisulfite E2NF	5'-TGGATGGTGATGTAAATGGTTATAAGTTT-3'	pDR-GFP
Bisulfite E2R	5'-GTTTGTGTTTTAGGATGTTGTTG-3'	pDR-GFP
Bisulfite E4R	5'-ACTTATACAACTCATCCATACCAAAAATAATCC-3'	pDR-GFP
Bisulfite E5R	5'-ACTTATACAACTCATCCATACCGAAAATAATCC-3'	pDR-GFP
Bisulfite E6R	5'-GGTTGTTATGAATAAAGGTGGTTATAAGA-3'	pDR-GFP
Bisulfite E7R	5'-CTCACTCATTAAACACCCCAAACTTTACAC-3'	pDR-GFP
Bisulfite E8R	5'-GAAGATTTTTPyGATTTGTAGTTTAAGTTTTAGG-3'	pDR-GFP
Bisulfite E9R	5'-GAAGATTTTTPyGATTTGTAGTTTAAGTTTTAGG-3'	pDR-GFP
ME-H19F	5'-GAGCCGCACCAGATCTTCAG-3'	hH19
ME-H19R	5'-TTGGTGGAACACACTGTGATCA-3'	hH19
ME-UBF	5'-CTCAGGGGTGGATTGTTGAC-3'	hUBE2B
ME-UBR	5'-TGTGGATTCAAAGACCACGA-3'	hUBE2B
mRNA-GF	5'-TTCACCACCATGGAGAAGGCT-3'	hGAPDH
mRNA-GR	5'-ACAGCCTTGGCAGCGCCAGT-3'	hGAPDH
mRNA-ACF	5'-AAAGCCATGCCAATCTCATC-3'	hB-ACTIN
mRNA-ACR	5'-GATCATTGCTCCTCCTGAGC-3'	hB-ACTIN
ME-ACpGR	5'-GCAAAGGCGAGGCTCTGT-3'	hB-ACTIN
ME-ACpGF	5'-GCGAAGCCGGTGAGTGAG-3'	hB-ACTIN
ME-PF	5'-GCAGTCCGACTCTCCAAAAG-3'	hp16
ME-PR	5'-AGCCAGTCAGCCGAAGGC-3'	hp16
ME-MGF	5'-GAGTCAGGCTCTGGCAGTGT-3'	hMGMT
ME-MGR	5'-GAGCTCCGCACTCTTCCGG-3'	hMGMT
mRNA-MYF	5'-CACCAGCAGCGACTCTGA-3'	hMYC
mRNA-MYR	5'-GATCCAGACTCTGACCTTTTGC-3'	hMYC
RNA-18SF	5'-TCCCCATGAACGAGGAATTC-3'	h18S
RNA-18SR	5'-GTGTACAAAGGGCAGGGACTT-3'	h18S

Supplementary Table S1. Complete list of DNA oligonucleotides used for PCR. On the left is shown the primer identification tag: 1. R, Rec1, Rec2, I-SceI, Bcg are the pDRGFP primers used for chromatin Immunoprecipitation analysis; 2. "Bisulfite" are the pDRGFP primers used for methylation analysis; 3. ME- primers are the primers used for control qPCR in the ChIP/MEDIP experiments; 4. mRNA-primers are the primers used for control qPCR in RT-qPCR experiments. On the centre is shown the DNA sequence; on the right are shown the specific genes or loci (human or mouse) corresponding to the specific primers.

Supplementary Movie. Time Lapse Microscopy of HDR. Live imaging of GFP repair by HDR in cells expressing inducible I-SceI (TetOn). The cells were induced with Doxycycline for 24 hours and then images were captured every 5 minutes covering a span of 30 hours. The signal is nuclear due to the presence of a NLS signal (aa 7-25) in GFP coding sequence. H and L represent High and Low GFP expressors as indicated in the text. There are 3 types of products of cell division: H-H; L-L; H-L. We interpret these 3 types of cells as derived from the first (square) or second (circle) cycle after HDR. Only in the first cycle after HDR, H and L are formed from the same cell. In subsequent cycles, H and L cells stably propagate in culture the H or L phenotype.

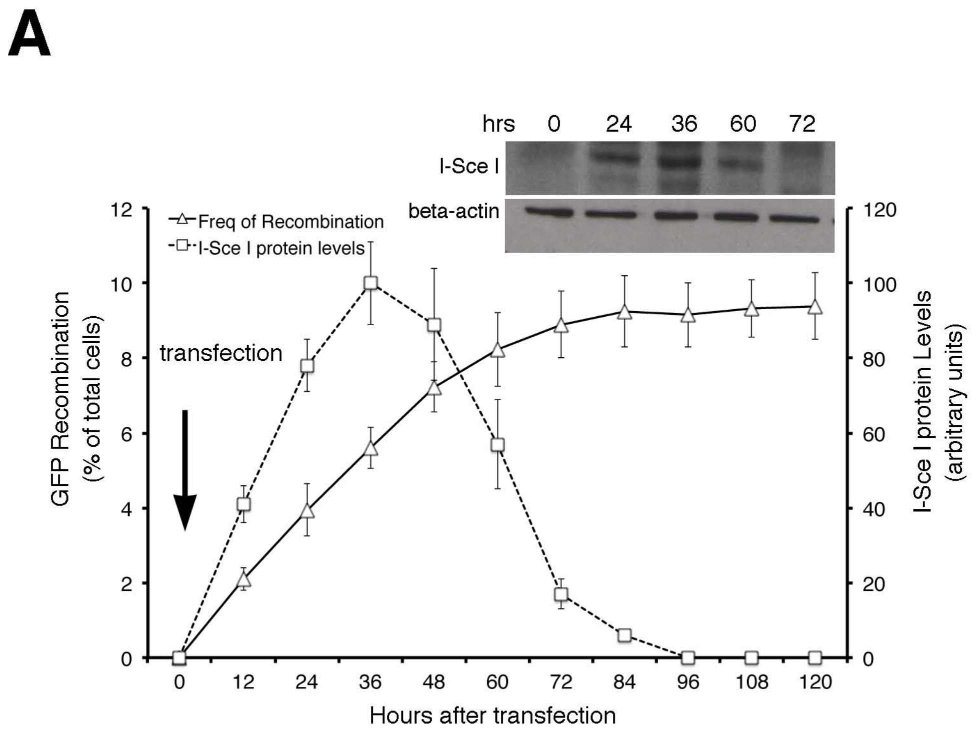

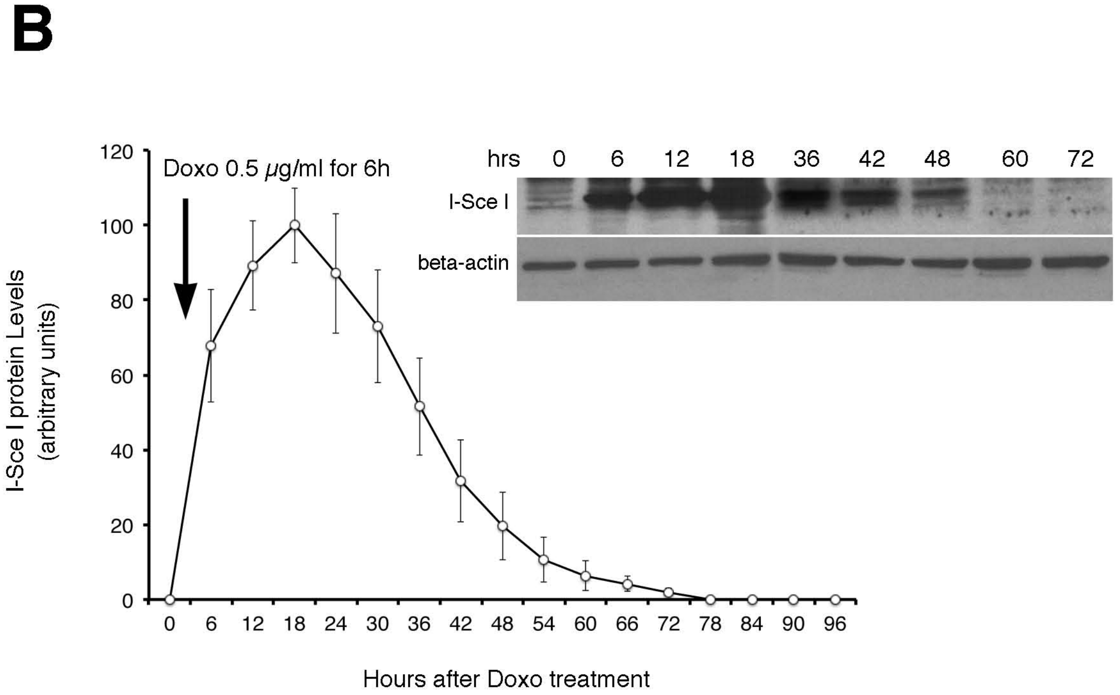

Supplementary Figure S1. Recombination and I-SceI levels. **A.** Recombination rate and I-SceI protein levels following I-SceI transfection. DRGFP Hela cells were transfected with I-SceI (arrow) and analyzed at the times indicated for: 1. GFP expression by FACS analysis: 2. I-SceI protein levels by immunoblot of total cell extracts (upper inset). The data are derived from independent experiments performed more than 10 times (FACS, n≥20) or 3 times in duplicate (immunoblot, n≥6). Data were plotted as mean ±SD. **B.** Hela cells clones expressing I-SceI under the control of TetOn regulatory sequences were characterized and the best responders were used for further studies (R.L and M.M., unpublished observations). The cells were induced with doxocyclin for 6h, as indicated, and total cell extracts used for immunoblot with anti-I-SceI specific antibodies.

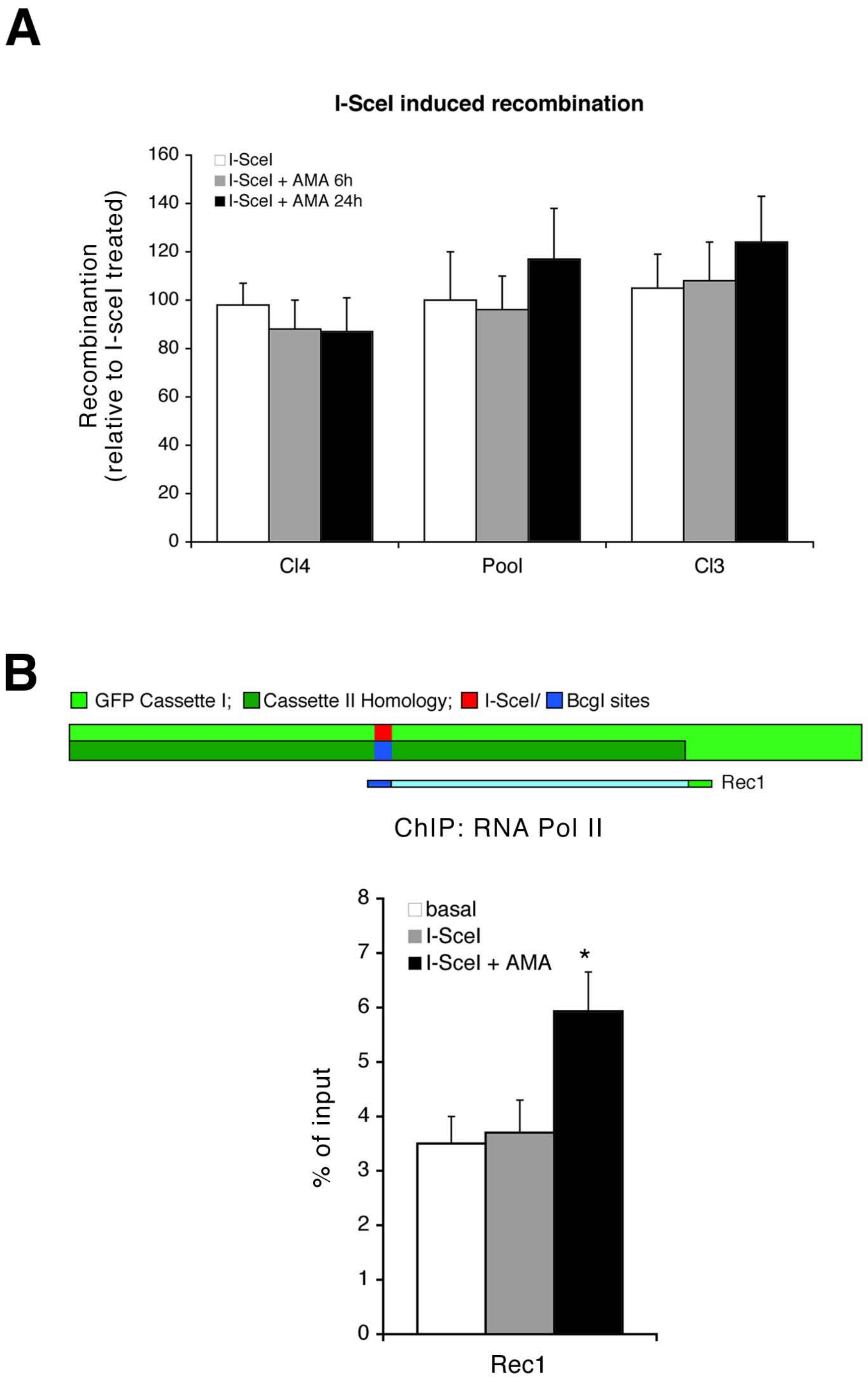

Supplementary Figure S2. Exposure of cells to α-amanitin during repair increases RNA polymerase II molecules on recombinant chromatin and does not alter the rate of homologous repair. A. Frequency of recombination in cells exposed to α-amanitin. The histogram shows the relative recombination frequency, deduced from the levels of recombinant GFP DNA, in cells exposed or not to α-amanitin. Specific primers, corresponding to the recombinant GFP, were used to amplify GFP in the total DNA extracted from the cell lines indicated. **B.** ChIP analysis with antibodies to the large subunit of RNA polymerase II in DRGFP cells, treated with α-amanitin for 24 h during repair. DRGFP cells were transfected with I-SceI and 12 h later exposed 24 h to 2.5 μM α-amanitin, The immunoprecipitated DNA was amplified by qPCR with the indicated primers: Bcg and Rec1, specific to the recombinant GFP cassette I. The ordinates represent the fraction of immunoprecipitated DNA relative to the input of chromatin-DNA present in the reactions. Differences between treatments were tested for statistical significance using Student's matched pairs *t* test: *p<0.01 as compared to the I-SceI.

Fig. S3

A

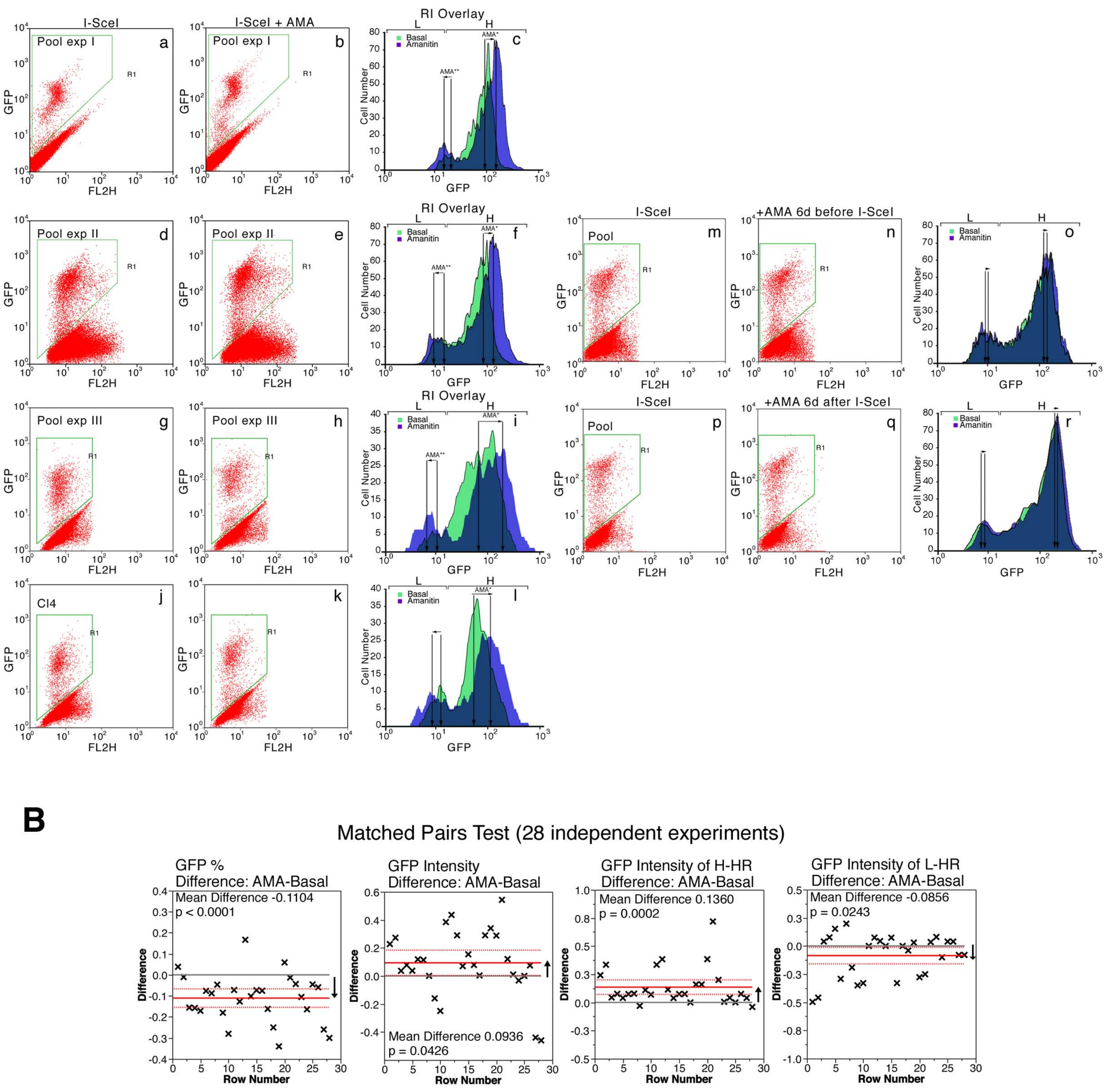

Supplementary Figure S3. Exposure to α-amanitin during repair, not before or after, amplifies and consolidates L and H clones. A. Cytofluorimetric analysis of cells exposed to α-amanitin in several independent experiments. The left and the center columns show dot plot scans of cells before (a, d, g, j, m, p) or after (b, e, h, k, n, q) α-amanitin treatment. The right column (c, f, i, l, m, o, r) shows the RI overlays. The first 3 rows show the dot plots of 3 (of 28) independent experiments, performed in the pool of DRGFP Hela clones, exposed to α-amanitin 12 h after I-SceI transfection. The rows j, k show clone 4 cells exposed to α-amanitin 12 h after I-SceI transfection. The rows m, n and p, q, show the pool of DRGFP Hela cells, exposed to α-amanitin before (36 h) or after (3 days) I-SceI transfection. Differences between treatments were tested for statistical significance using Student's matched pairs *t* test: *p<0.001, **p<0.05. The panel **B** shows the difference plot of GFP fluorescence intensity or percentage of GFP positive cells in each pairs of experiments with or without α-amanitin (matched pairs test). The difference mean is indicated by the red line and the expected value for the null hypothesis, set to 0, is indicated by a black line (SD is indicated by red dashed lines).

Fig. S4

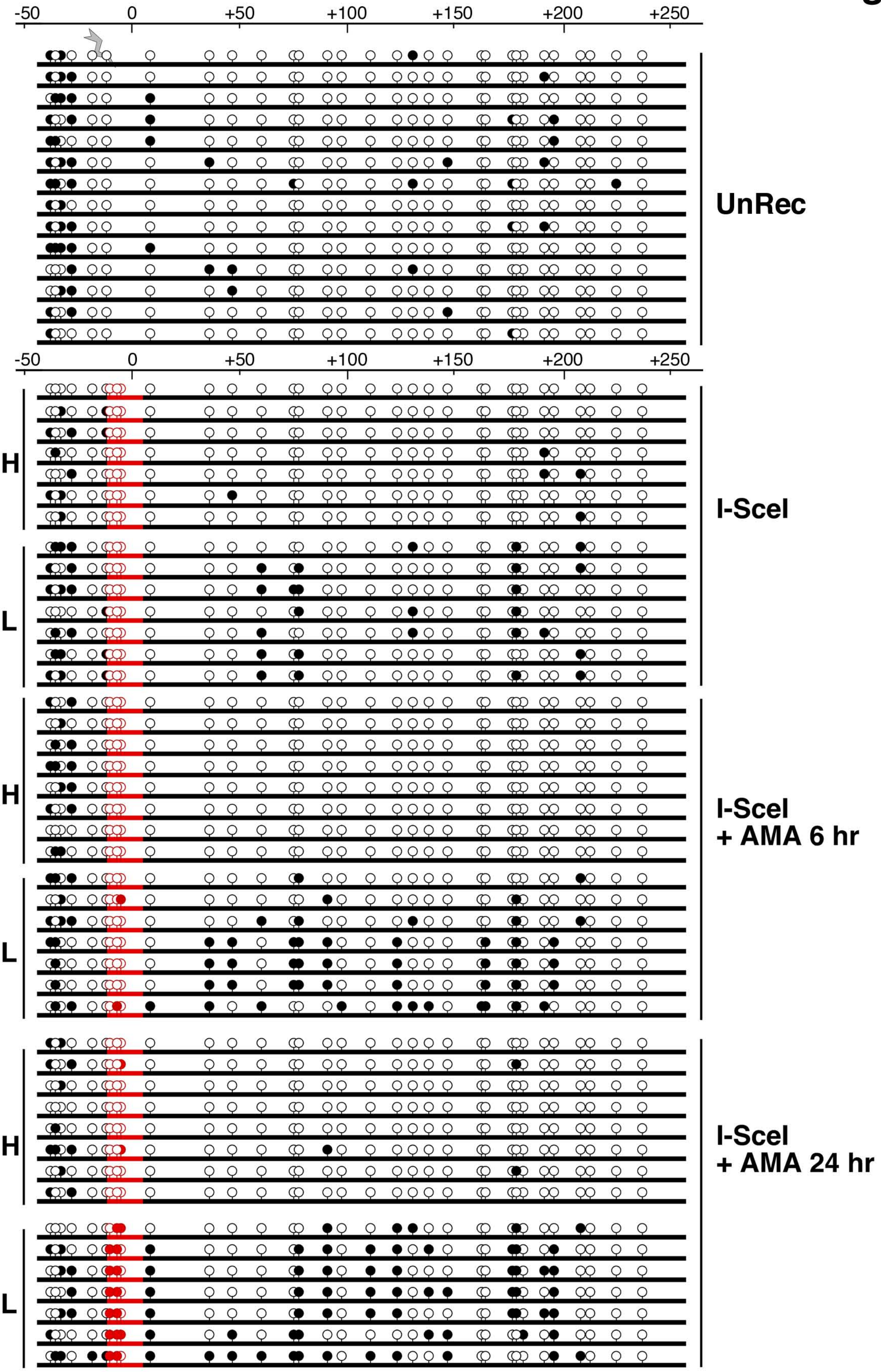

Supplementary Figure S4. DNA Methylation of the GFP repaired segment in α-amanitin-exposed cells. Methylation analysis of the non-recombinant (UnRec) and recombinant (I-SceI) GFP DNA isolated from cells exposed to α-amanitin (2.5 μM). Where indicated, DRGFP clone 4 was transfected with I-SceI and after 24 h was treated with α-amanitin for 6 h or 24 h. At the end of incubation, the cells were **split** and cultured for at least 5 days, before sorting them into L and H cells, as indicated in Materials and Methods. DNA from sorted cells was treated with bisulfite. 48 h treatment with α-amanitin did not significantly alter the methylation pattern observed in cells exposed to the drug for 6 h or 24 h. The sequence coordinates of GFP, relative to the *I-SceI* site (0 or red in recombinant molecules) are shown on the top. The methylated CpGs are indicated by black circles.

Fig. S5

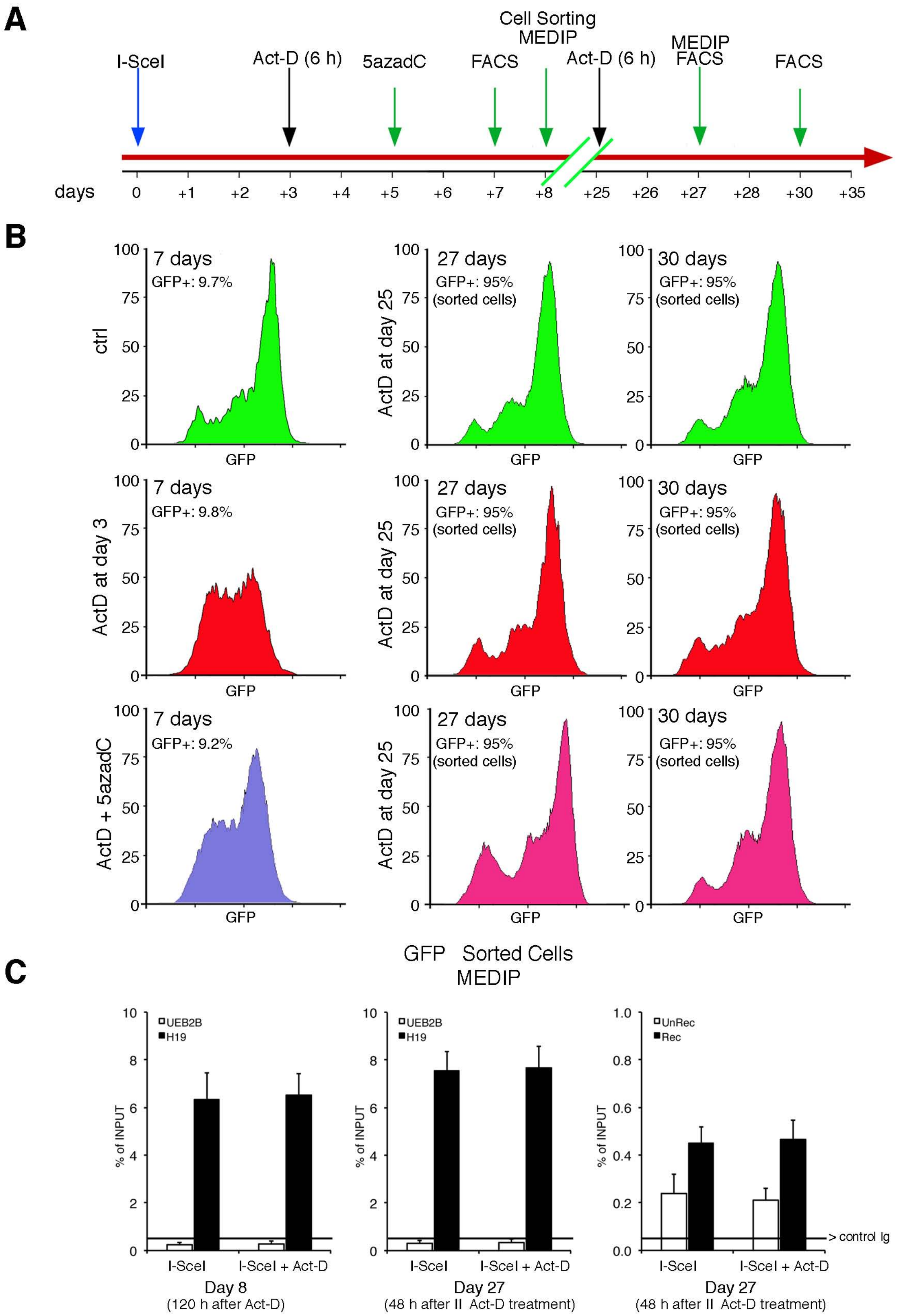

Supplementary Figure S5. Act-D effects on GFP expression occur only in a definite time window after after repair. 5azadC eliminates Act-D effects on GFP expression. A. Time frame of Act-D treatments after I-SceI expression. **B.** Cells were treated with Act-D as described in Fig. 7 at day 3 after I-SceI transfection. Two days later (day 5) an aliquot of cells was treated with 5azadC and analyzed at day 7 (FACS) and at day 8 for MEDIP. Twenty five (25) days later, the cells were exposed 6 h to Act-D and analyzed for MEDIP and FACS at day 27 and at day 30, as shown in the second or third column. Cells expressing CMV-EGFP treated for 6 h with Act-D did not modify GFP expression 48 h after treatment. **C.** MEDIP analysis in UEB2B, H19 and non-recombinant and recombinant GFP 3' of the *I-SceI* site. The days and the time of Act-D treatment is shown below the histograms.

Fig. S6

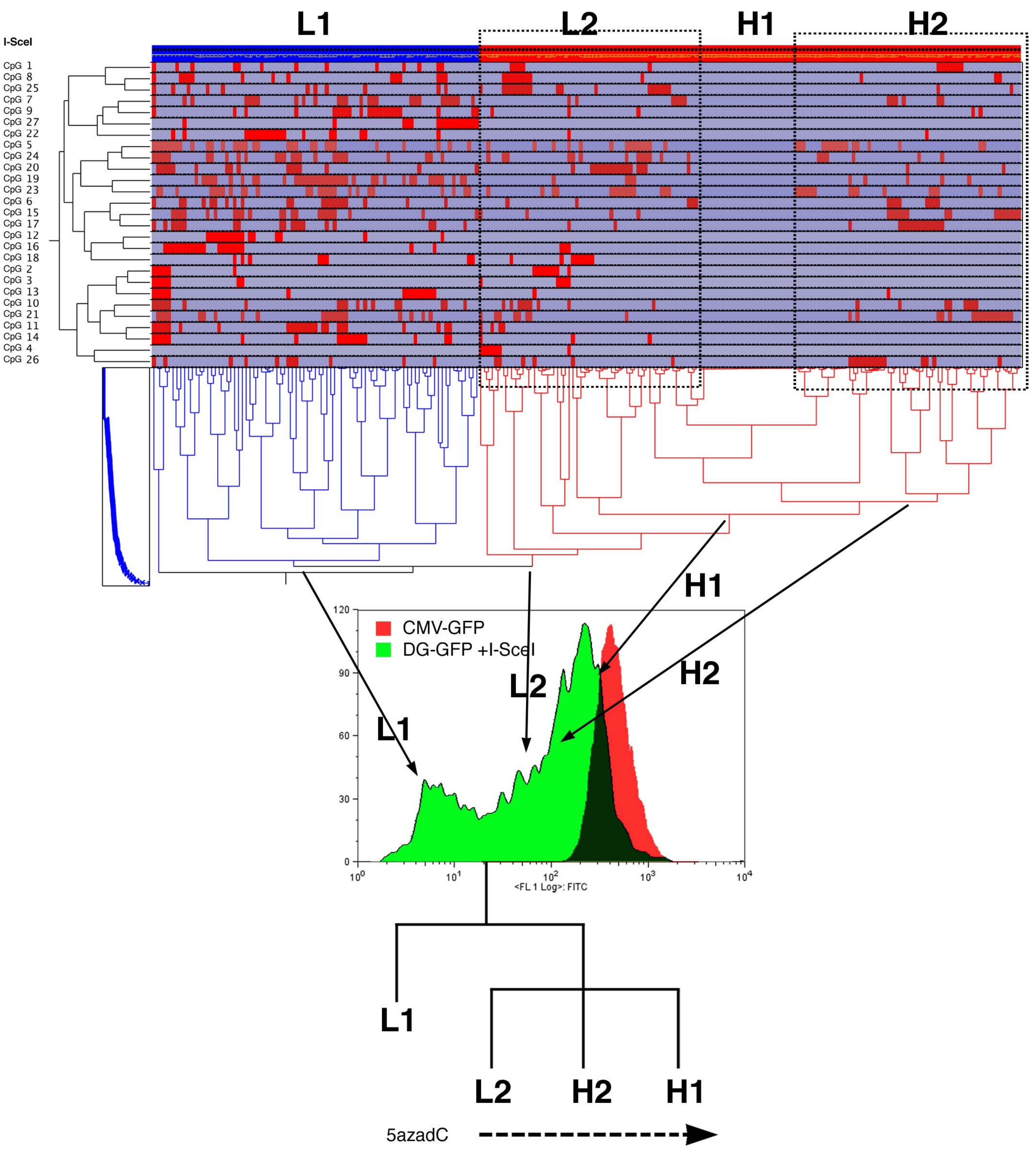

Supplementary Figure S6. Hierarchical cluster analysis of methylated GFP DNA molecules after homologous repair in ES and Hela cells. Bisulfite GFP sequences from I-SceI transfected and sorted HeLa and ES DRGFP cells were cloned, sequenced and analyzed by clustal analysis. The upper panel shows the dendrogram with the color map of the two-way hierarchical clustering (Ward's criterion) performed on CpG 1 to 27 at 3' end of *I-SceI* site. Clustal analysis was performed using the *JMP Statistical Discovery™* software by SAS, Statistical Analysis Software. On the left are indicated the 3’ end CpGs, relative to the *I-SceI* site (0). Clustal analysis shows that all methylated molecules, whether derived from ES or Hela cells, can be clustered into 2 main groups: the first, (blue) corresponds to hypermethylated L (L1) cells, expressing low GFP levels; the second, (red) is distributed over a wide range of expression and includes GFP molecules with methylation profiles similar to methylated (L2) and undermethylated (H2) clones. The middle panel shows the FACS analysis of sorted cells (green) from which the GFP bisulfite sequences were derived (arrows). The red overlay shows the fluorescence plot of undamaged CMV-GFP expressing cells. The lower panel shows a simplified scheme of L and H populations derived from the clustal analysis. The horizontal arrow indicates the shift of cell populations after the 5azadC treatment.

A

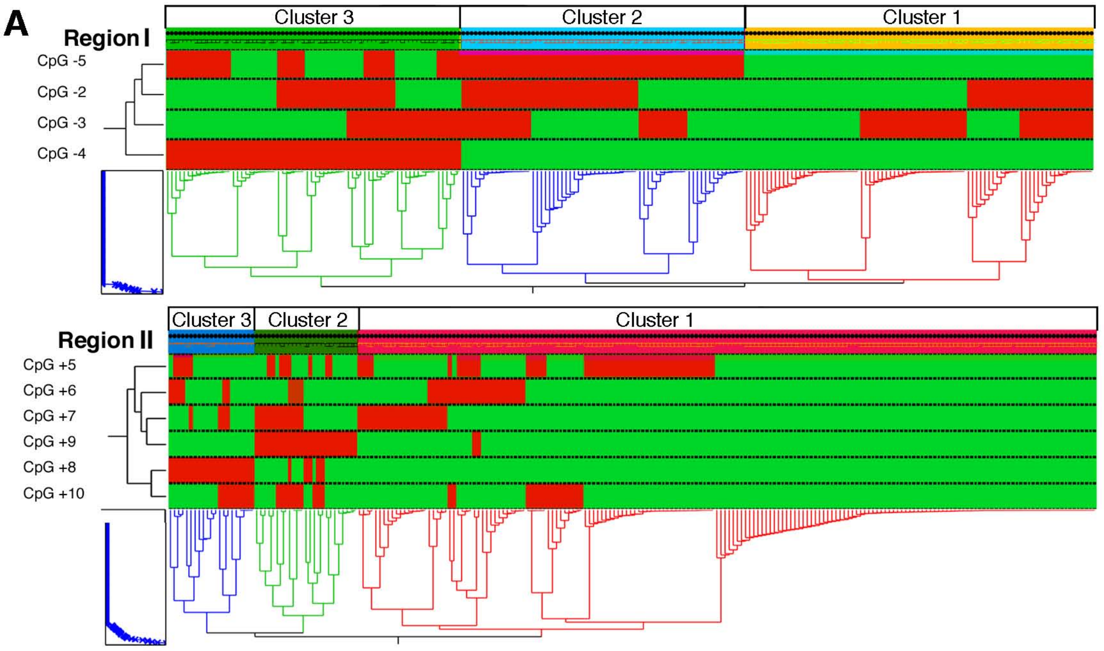

B

Rec by Region I

Contingency Table

Cluster Region I (Count / Expected)	Rec	UnRec	
Cl 1	44 / 51.5265	41 / 33.4735	85
Cl 2	47 / 43.646	25 / 28.354	72
Cl 3	46 / 41.8274	23 / 27.1726	69
	137	89	226

Tests

Source	DF	-LogLike	RSquare (U)
Model	2	2.23972	0.0148
Error	223	149.27516	
C. Total	225	151.51489	
N	226		

Test	ChiSquare	Prob>ChiSq
Likelihood Ratio	4.479	0.1065
Pearson	4.503	0.1052

Expression by Region I

Contingency Table

Cluster Region I (Count / Expected)	High	Low	
Cl 1	34 / 28.9635	28 / 33.0365	62
Cl 2	15 / 16.8175	21 / 19.1825	36
Cl 3	15 / 18.219	24 / 20.781	39
	64	73	137

Tests

Source	DF	-LogLike	RSquare (U)
Model	2	1.545166	0.0163
Error	134	93.120165	
C. Total	136	94.665330	
N	137		

Test	ChiSquare	Prob>ChiSq
Likelihood Ratio	3.090	0.2133
Pearson	3.080	0.2144

Rec by Region II

Contingency Table

Cluster Region II (Count / Expected)	Rec	UnRec	
Cl 1	97 / 109.115	83 / 70.885	180
Cl 2	21 / 15.1549	4 / 9.84513	25
Cl 3	19 / 12.7301	2 / 8.26991	21
	137	89	226

Tests

Source	DF	-LogLike	RSquare (U)
Model	2	9.69731	0.0640
Error	223	141.81758	
C. Total	225	151.51489	
N	226		

Test	ChiSquare	Prob>ChiSq
Likelihood Ratio	19.395	<.0001*
Pearson	16.982	0.0002*

Expression by Region II

Contingency Table

Cluster Region II (Count / Expected)	High	Low	
Cl 1	58 / 45.3139	39 / 51.6861	97
Cl 2	5 / 9.81022	16 / 11.1898	21
Cl 3	1 / 8.87591	18 / 10.1241	19
	64	73	137

Tests

Source	DF	-LogLike	RSquare (U)
Model	2	13.858953	0.1464
Error	134	80.806377	
C. Total	136	94.665330	
N	137		

Test	ChiSquare	Prob>ChiSq
Likelihood Ratio	27.718	<.0001*
Pearson	24.207	<.0001*

C

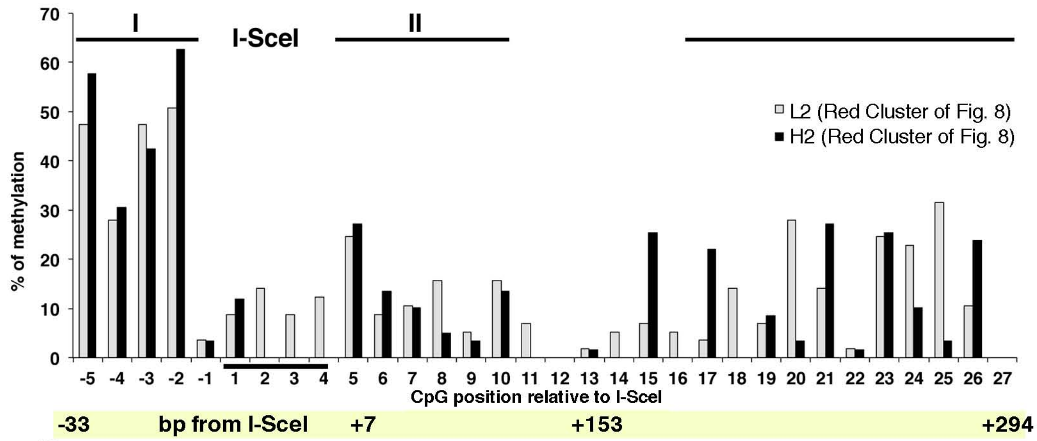

D

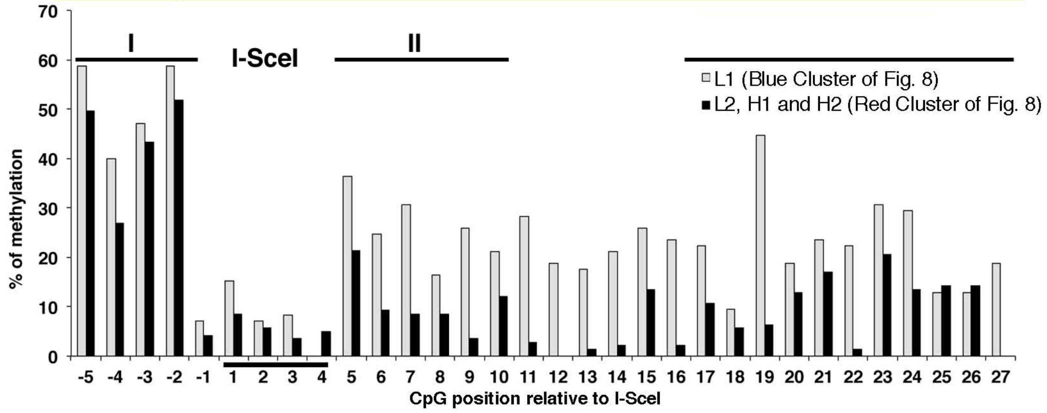

Supplementary Figure S7. Selective methylation at the 3' of the DSB discriminates recombinant versus non-recombinant GFP molecules. A. Statistical analysis (cluster analysis) of the methylation profiles upstream (Region I) and downstream (Region II) of the DSB in recombinant and non-recombinant GFP, along the direction of transcription. On the left are indicated the CpGs analyzed, (-) and (+) relative to the *I-SceI/BcgI* site. CpGs n. 1, 2, 3 and 4 have been excluded from the analysis because are specific to the recombinant *BcgI* site and are not present in the non-recombinant *I-SceI* sequence. Molecules carrying similar methylation profiles (same position of methylated CpGs) are grouped in clusters shown by different colours. **B.** The selective distribution of clusters in the recombinant and the non-recombinant clones and in L (Low) and H (High) cells has been tested by using contingency tables and by determining χ^2. The contingency tables show the distribution of recombinant sequences and of L and H cells in the methylation clusters of Region I and Region II (Rec or Expression by Region I or II). The methylation profiles of CpGs upstream of the I-SceI site, 5' to the DSB in the direction of transcription (region I), were not modified by repair and did not discriminate recombinant versus non-recombinant GFP (χ^2 4.47; p=0.1) or L versus H cells (χ^2 3.09; p=0.21). Conversely, methylation profiles of region II at the 3' end of *I-SceI*, discriminated recombinant versus non-recombinant molecules (χ^2 19.39; p<0.0001) and L from H cells (χ^2 27.718; p<0.0001). We extended the methylation analysis 500 bp upstream of *I-SceI*. Here there are 22 CpGs of which only the 5 CpGs adjacent to *I-SceI* (-33 bp) are methylated (data not shown) and the methylation status of methylated or unmethylated CpGs does not change in recombinant cells. **C** and **D.** Methylation frequency (expressed as % of the total molecules) of the CpGs upstream and downstream of *I-SceI* in L and H clones. The data were derived from DRGFP Hela cells (approximately 500 sequences) sorted as H and L cells as shown in Supplementary Figure S6. Cluster analysis based on the similarity of methylation profiles identifies 2 main clusters, color coded, which include L1 (blue cluster in Supplementary Figure S6), L1, H1 and H2 (red cluster in Supplementary Figure S6) clones. The location of CpGs in these 2 clusters is shown. CpGs 1 to 4 (marked by a black line) correspond to the *BcgI* site generated by recombination. These CpGs, not included in the statistical analysis shown in A and B, are not methylated in H2 and are able to distinguish the H2 from the L2 cells (see also, Supplementary Figure S6).

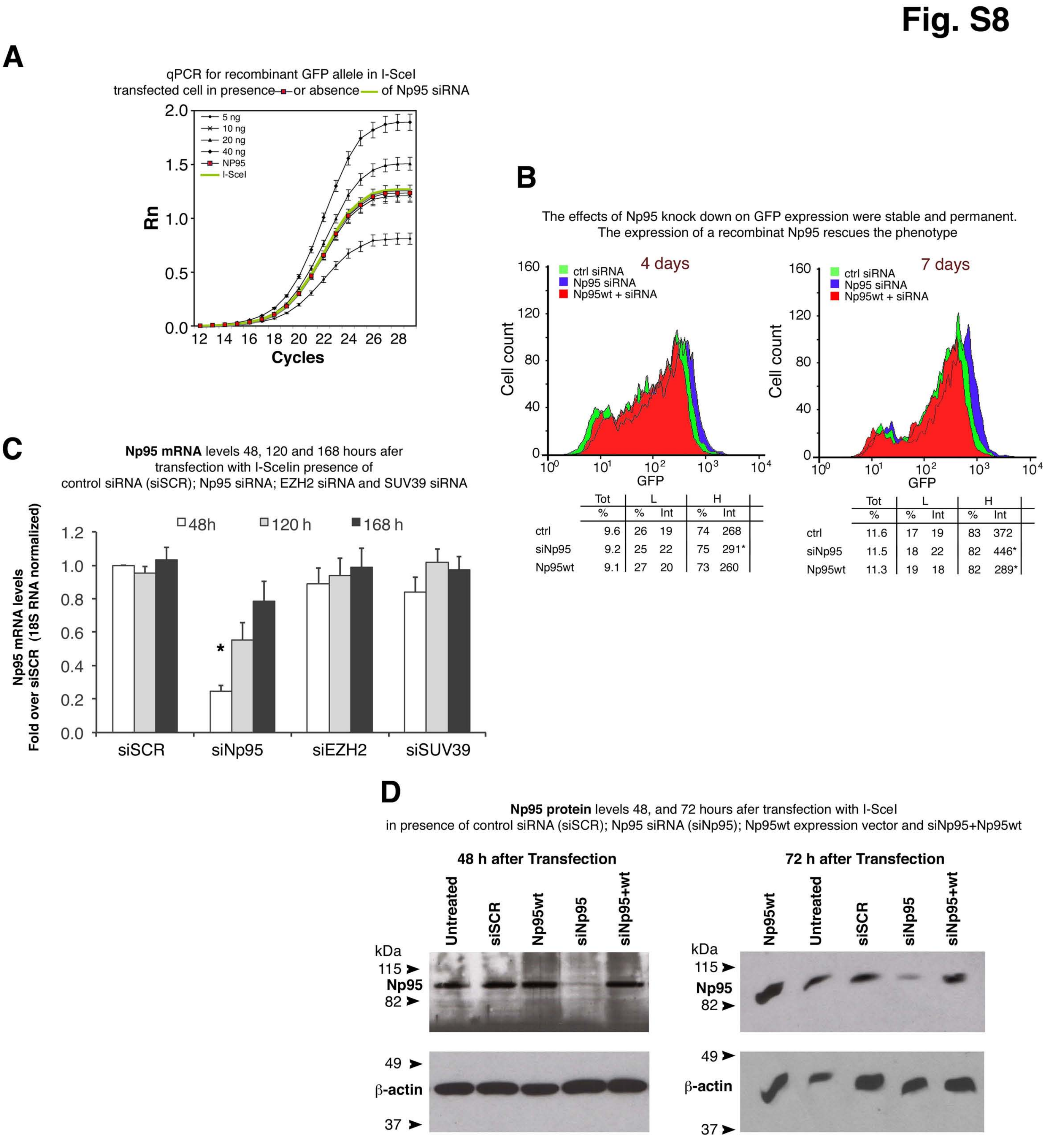

4 days:

	Tot	L		H	
	%	%	Int	%	Int
ctrl	9.6	26	19	74	268
siNp95	9.2	25	22	75	291*
Np95wt	9.1	27	20	73	260

7 days:

	Tot	L		H	
	%	%	Int	%	Int
ctrl	11.6	17	19	83	372
siNp95	11.5	18	22	82	446*
Np95wt	11.3	19	18	82	289*

Supplementary Figure S8. Frequency of recombination and Np95 expression in Np95-silenced cells. Cells were transfected with I-SceI and Np95 siRNA and analyzed at 48, 72, 120 and 168 h after transfection. **A.** Recombination rate in silenced cells. The DNA was extracted from DRGFP cells 72 h after transfection. The rate of recombination was measured by determining the amount of recombinant GFP generated by DSB repair (I-SceI) at different DNA concentrations. The green curve indicates the amount of repaired GFP in control I-SceI transfected and sorted cells (100 ng DNA). A single copy gene has been used as internal standard. The amount of repaired GFP in the pool of clones corresponds approximately to 10% of the control DNA. Specific GFP primers (Bcg and Rec1) were used to determine the amount of recombinant GFP gene in the cell population by qPCR. **B**. The effects of Np95 knock-down on GFP expression were visible 7 days after the damage. The cells were transfected with siRNAs (control or siNp95) with or without an expression vector encoding the mouse Np95 gene. The samples were collected at 4 and 7 days and analyzed by cytofluorimetry. The columns below the fluorescence plot show: i. the number of GFP^{+} cells (Tot, expressed as percentage of cells); ii. the mean fluorescence intensity (Int.); and, iii. Percentage of L and H cells in GFP^{+} cells. *p<0.01 for *t* value (matched pair test) relative to the cells treated with control scramble siRNA (CTRL). **C.** RNA analysis in Np95-silenced cells 48, 120 and 168 hours after transfection. The mRNA levels of Np95 were determined by qPCR in cells transfected with I-SceI and Np95 siRNA; as control scrambled (siSCR), EZH2 and SUV39 siRNAs were used. Data are shown as fold increase over control (mean ±SD). *p<0.01, paired *t* test. **D.** Np95 protein levels in cell transfected with Np95 siRNA and/or Np95wt expression vector. Western Blot analysis on total protein extracts 48 and 72 h after transfection. Gel loading was normalized for β-actin levels.

Fig. S9

A

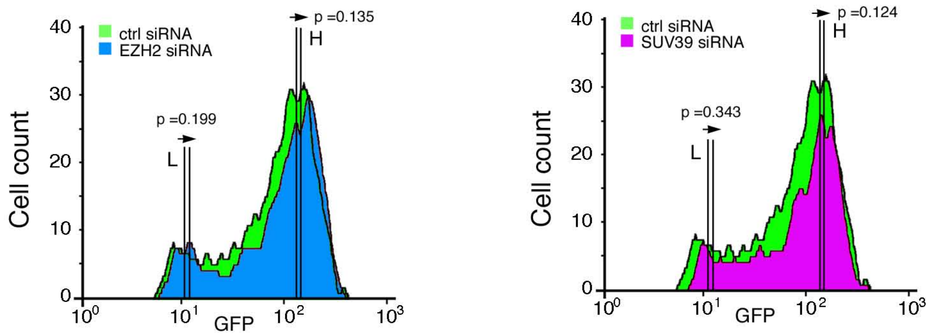

	Tot	L		H	
	%	%	Int	%	Int
ctrl	4.2	20	14	80	250
siEZH2	3.8	20	15	80	290

	Tot	L		H	
	%	%	Int	%	Int
ctrl	4.2	20	14	80	250
siSUV39	3.1*	19	15	81	280

B

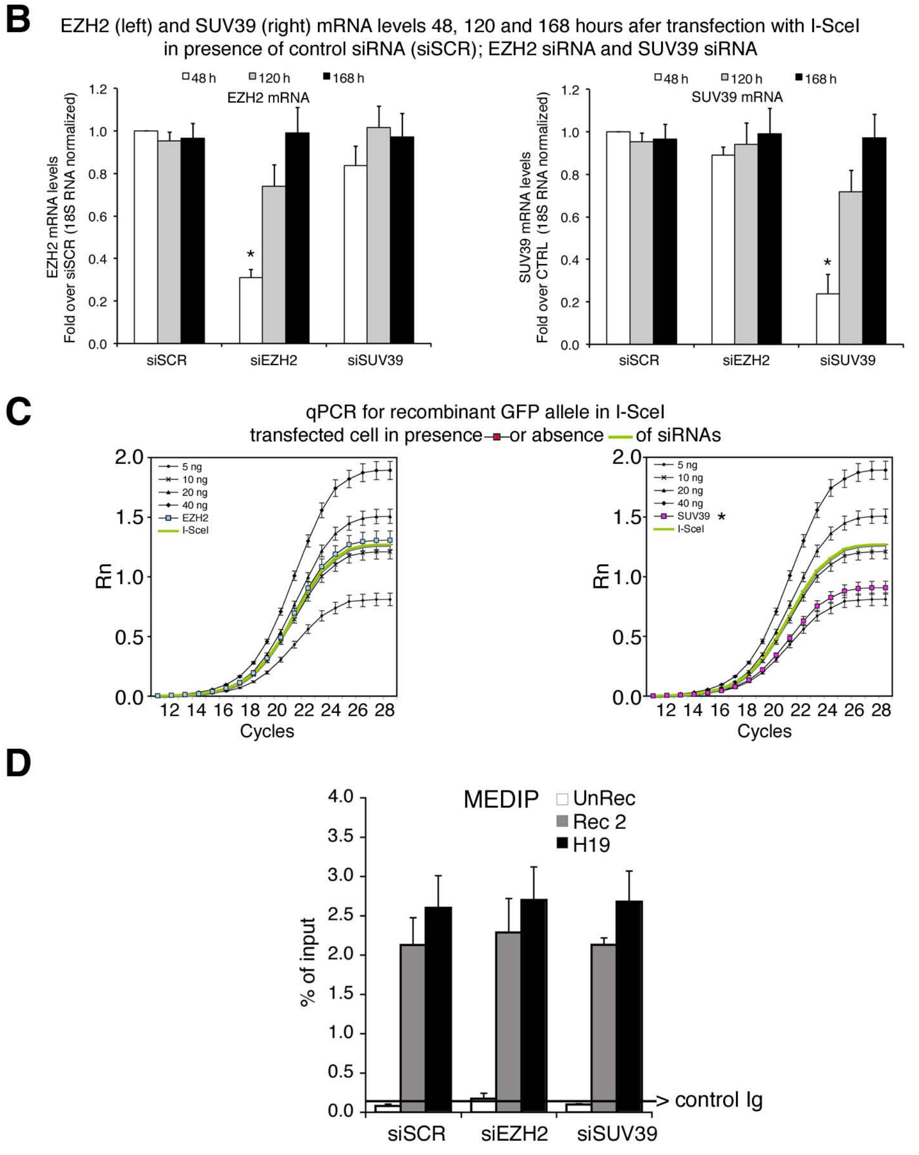

Supplementary Figure S9. Expression analysis, frequency of recombination, and GFP methylation in EZH2 or SUV39-silenced cell **A.** DRGFP cells (pool of clones; clones 3 and 4 are not shown) were transiently transfected with siRNA pools targeting EZH2 or SUV 39 control scrambled siRNA (ctrl) and the I-SceI expression vector. Six days later the cells were subjected to FACS analysis as described in Fi 1. The arrows indicate the shift in silenced cells of GFP fluorescence intensity. The columns below the panels show: i. the number of GFI cells (Tot, expressed as percent of cells); ii. the median fluorescence intensity (Int.); and, iii. percentage of L and H cells in GFP$^+$ cells. FAC analysis was performed in triplicate in at least 3 experiments (n≥9). Differences between treatments were tested for statistical significan using Student's matched pairs *t* test: *p <0.01 as compared to the each control. All the samples in independent experiments were treated with µM 5azadC for 1 day (48 h after I-SceI) and the difference of fluorescence intensity was used to quantify methylation-dependent changes GFP expression. **B.** RNA analysis in EZH2 or SUV39-silenced cells 48, 120 and 168 h after transfection. mRNAs levels were determined b qPCR in cells transfected with I-SceI and siRNAs; as control, scrambled RNAs (siSCR) were used. Data are shown as fold increase ov control (mean ±SD). *p<0.01, paired *t* test. **C.** Recombination rate in silenced cells. The DNA was extracted from DR-GFP cells 72 h aft transfection. The rate of recombination was measured as described in legend of Fig. S5 (panel A). *p<0.01, paired *t* test. **D.** 5mC content recombinant GFP in cells silenced for EZH2 and SUV39. The samples, in A, B and C, were processed as described in Materials and Methods

Fig. S10

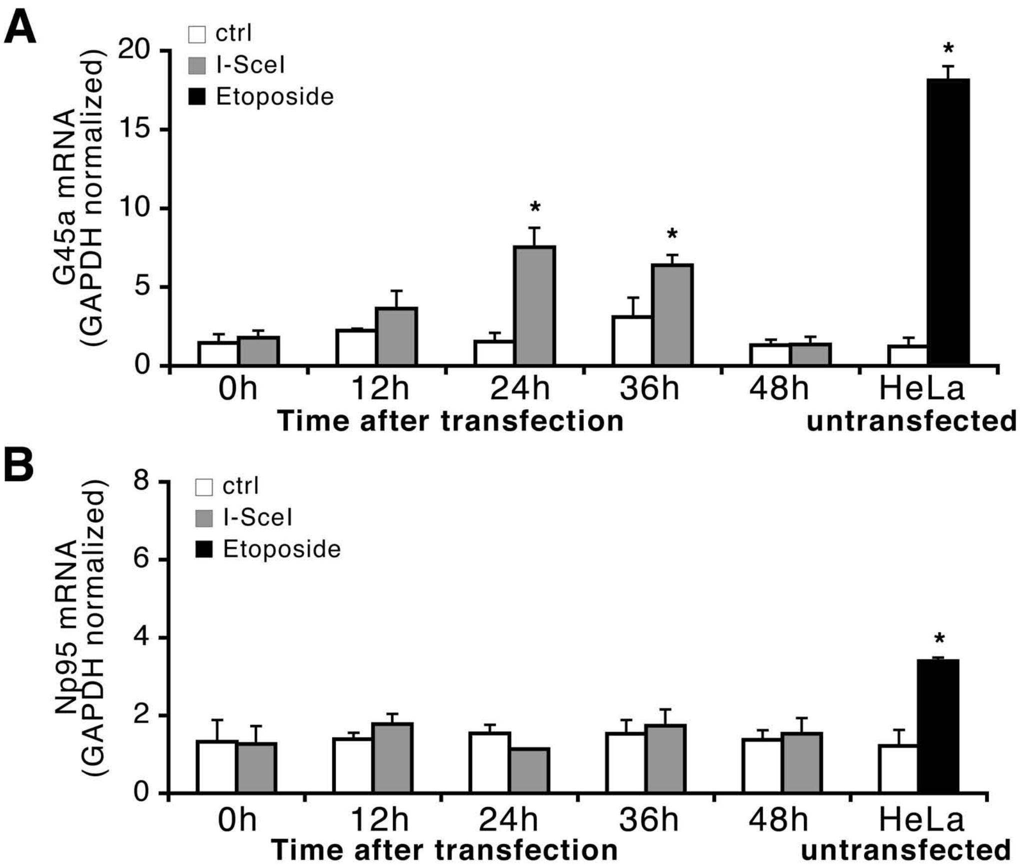

Supplementary Figure S10. GADD45a is transiently induced by DNA damage. mRNA levels of GA45a (**A**) or Np95 (**B**) in HeLa cells exposed to etoposide (black) or transfected with I-SceI (grey). Cells were treated with etoposide (1µM for 2 h) or transfected with I-SceI for 12, 24 and 36 h. The data are derived from at least 3 experiments in triplicate (n≥9). Differences between treatments were tested for statistical significance using Student's matched pairs *t* test: *p <0.01 as compared to the each control.

Fig. S11

A

GadStat: Matched Pairs

Matched Pairs Expressor=H %

Difference: GADD45 2dd-ctrl 2dd				Difference: GADD45 4dd-ctrl 4dd				Difference: GADD45 7dd-ctrl 7dd			
GADD45 2dd	36.1444	t-Ratio	-8.7598	GADD45 4dd	73.4438	t-Ratio	0.446918	GADD45 7dd	76.8438	t-Ratio	-0.11894
ctrl 2dd	45.1688	DF	15	ctrl 4dd	73.3063	DF	15	ctrl 7dd	76.9375	DF	15
Mean Difference	-9.0244	Prob > |t|	<.0001*	Mean Difference	0.1375	Prob > |t|	0.6613	Mean Difference	-0.0938	Prob > |t|	0.9069
Std Error	1.0302	Prob > t	1.0000	Std Error	0.30766	Prob > t	0.3307	Std Error	0.78822	Prob > t	0.5465
Upper95%	-6.8285	Prob < t	<.0001*	Upper95%	0.79327	Prob < t	0.6693	Upper95%	1.5863	Prob < t	0.4535
Lower95%	-11.22			Lower95%	-0.5183			Lower95%	-1.7738		
N	16			N	16			N	16		
Correlation	0.53652			Correlation	0.9807			Correlation	0.9063		

Matched Pairs Expressor=H Int.

Difference: GADD45 2dd-ctrl 2dd				Difference: GADD45 4dd-ctrl 4dd				Difference: GADD45 7dd-ctrl 7dd			
GADD45 2dd	102.344	t-Ratio	-7.37165	GADD45 4dd	205.125	t-Ratio	-5.6232	GADD45 7dd	284.938	t-Ratio	-4.14594
ctrl 2dd	128.75	DF	15	ctrl 4dd	253.5	DF	15	ctrl 7dd	315.5	DF	15
Mean Difference	-26.406	Prob > |t|	<.0001*	Mean Difference	-48.375	Prob > |t|	<.0001*	Mean Difference	-30.563	Prob > |t|	0.0009*
Std Error	3.58214	Prob > t	1.0000	Std Error	8.60275	Prob > t	1.0000	Std Error	7.37166	Prob > t	0.9996
Upper95%	-18.771	Prob < t	<.0001*	Upper95%	-30.039	Prob < t	<.0001*	Upper95%	-14.85	Prob < t	0.0004*
Lower95%	-34.041			Lower95%	-66.711			Lower95%	-46.275		
N	16			N	16			N	16		
Correlation	0.49817			Correlation	0.10341			Correlation	0.51033		

Matched Pairs Expressor=L %

Difference: GADD45 2dd-ctrl 2dd				Difference: GADD45 4dd-ctrl 4dd				Difference: GADD45 7dd-ctrl 7dd			
GADD45 2dd	64.0875	t-Ratio	5.92993	GADD45 4dd	26.2813	t-Ratio	-1.28666	GADD45 7dd	22.25	t-Ratio	0.098342
ctrl 2dd	55.1125	DF	15	ctrl 4dd	27.0625	DF	15	ctrl 7dd	22.1875	DF	15
Mean Difference	8.975	Prob > |t|	<.0001*	Mean Difference	-0.7813	Prob > |t|	0.2177	Mean Difference	0.0625	Prob > |t|	0.9230
Std Error	1.51351	Prob > t	<.0001*	Std Error	0.60719	Prob > t	0.8911	Std Error	0.63554	Prob > t	0.4615
Upper95%	12.201	Prob < t	1.0000	Upper95%	0.51295	Prob < t	0.1089	Upper95%	1.41711	Prob < t	0.5385
Lower95%	5.74903			Lower95%	-2.0755			Lower95%	-1.2921		
N	16			N	16			N	16		
Correlation	0.55897			Correlation	0.44183			Correlation	0.67615		

Matched Pairs Expressor=L Int.

Difference: GADD45 2dd-ctrl 2dd				Difference: GADD45 4dd-ctrl 4dd				Difference: GADD45 7dd-ctrl 7dd			
GADD45 2dd	9.99438	t-Ratio	-3.71876	GADD45 4dd	10.2006	t-Ratio	-3.76046	GADD45 7dd	11.1188	t-Ratio	-1.22122
ctrl 2dd	11.7313	DF	15	ctrl 4dd	11.8	DF	15	ctrl 7dd	11.9438	DF	15
Mean Difference	-1.7369	Prob > |t|	0.0021*	Mean Difference	-1.5994	Prob > |t|	0.0019*	Mean Difference	-0.825	Prob > |t|	0.2409
Std Error	0.46706	Prob > t	0.9990	Std Error	0.42531	Prob > t	0.9991	Std Error	0.67556	Prob > t	0.8796
Upper95%	-0.7414	Prob < t	0.0010*	Upper95%	-0.6928	Prob < t	0.0009*	Upper95%	0.61491	Prob < t	0.1204
Lower95%	-2.7324			Lower95%	-2.5059			Lower95%	-2.2649		
N	16			N	16			N	16		
Correlation	0.8173			Correlation	0.86972			Correlation	0.69832		

B

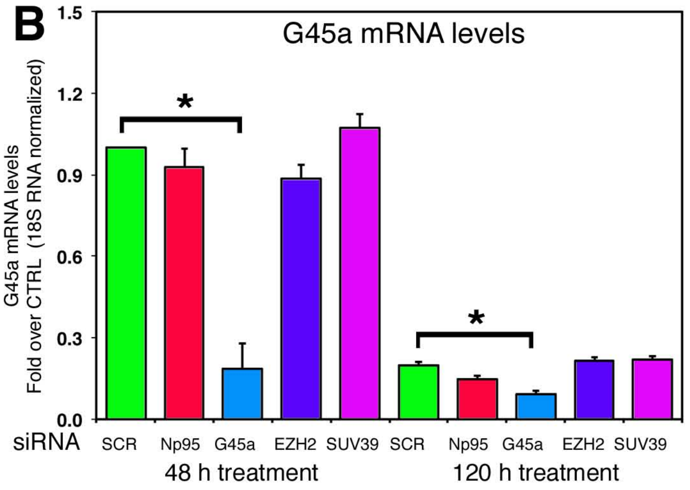

C

Matched Pairs GFP+

Difference: G45a -ctrl

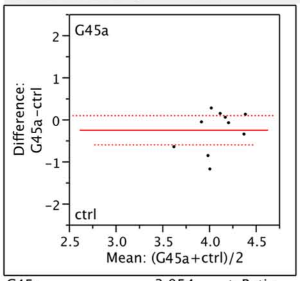

G45a	3.954	t-Ratio	-1.69389
ctrl	4.214	DF	9
Mean Difference	-0.26	Prob > |t|	0.1245
Std Error	0.15349	Prob > t	0.9377
Upper95%	0.08722	Prob < t	0.0623
Lower95%	-0.6072		
N	10		
Correlation	-0.0782		

D

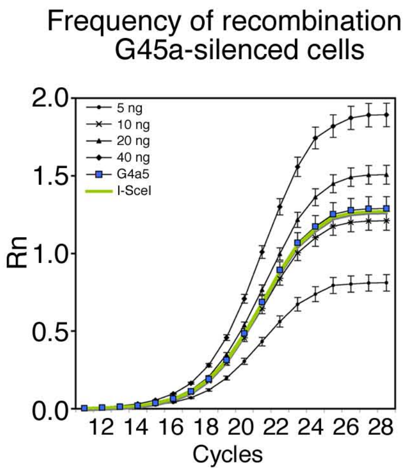

E

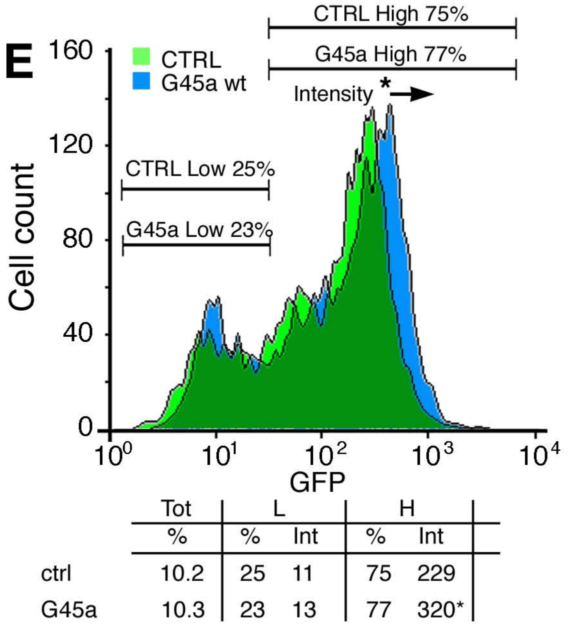

	Tot	L		H	
	%	%	Int	%	Int
ctrl	10.2	25	11	75	229
G45a	10.3	23	13	77	320*

Supplementary Figure S11. GFP expression in GA45a-silenced cells. A. Fluorescence data of cells treated with siRNA targeting GA45a, derived from 5 experiments in triplicate (n=15), were compared in matched pairs *t* test. Difference between treatments (siG45a vs ctrl) for fluorescence intensity (Expressor=L-int. or H-int.) and for cell number (Expressor=L% or H%) were tested for statistical significance using Student's matched pairs t test. 2dd, 4dd and 7dd indicate days after transfection (see Fig. 7C) **B.** RNA analysis in GA45a-silenced cells. In all transfections performed with the various siRNAs (indicated on the abscissa) mRNA levels of GA45a were determined by qPCR. Data are shown as fold increase over control (mean ±SD). *p<0.01, paired *t* test. **C.** Matched pairs test comparing GFP positive in control and GA45a-silenced cells, which shows that the fraction of GFP positive cells is not modified by the siRNA targeted to GA45a. **D.** Recombination rate in GA45a-silenced cells. The DNA was extracted from DRGFP cells 72 h after transfection. The rate of recombination was measured as described in legend of Supplementary Figure S8 (panel A). **E.** Overexpression of G45a increases fluorescence intensity of H population. DRGFP cells (pool of clones; clones 3 and 4 are not shown) were transiently transfected with G45a and the I-SceI expression vectors. Six (6) and fourteen (14) days later the cells were subjected to FACS analysis as described in Fig. 1. The overlay shows the experiment at day 6. At day 14 GFP expression was comparable between ctrl and G45a transfected cells. The arrows indicate the shift in G45a overexpressing cells of GFP fluorescence intensity. The columns below panels show: i. the number of GFP^+ cells (Tot, expressed as percent of cells); ii. the median fluorescence intensity (*Int.*); and, iii. % of L and H cells in GFP^+ cells. FACS analysis was performed in at least 3 experiments in triplicate. *p<0.01, paired *t* test.

Fig. S12

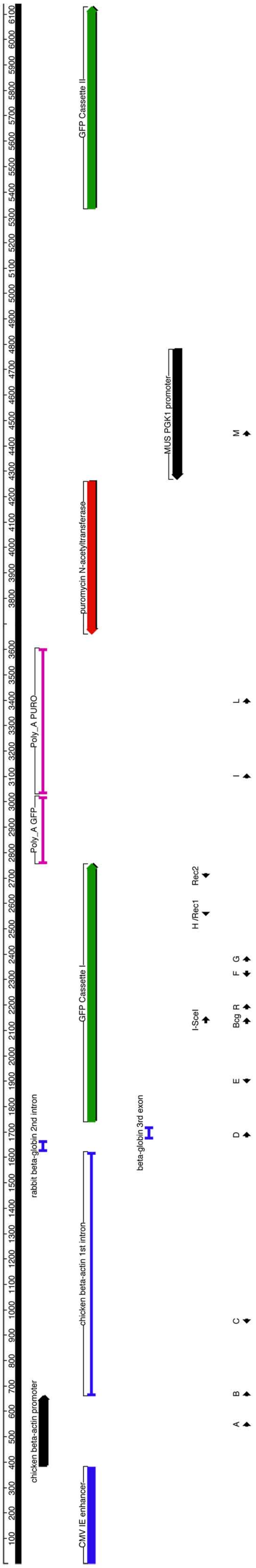

Supplementary Figure S12. GFP primers used to analyze recombination, methylation and DNA chromatin domains. Location of the DRGFP primers used. The direction of transcription is indicated by arrows. The arrows indicate: 1. green, the I and II GFP cassettes; 2. red, the puromycin resistance gene; 3. black and blue, the promoters and enhancers, respectively.
